# Single‐Sensor Vibration Sensing in Anisotropic Composites Enabled by Disordered Metasurfaces

**DOI:** 10.1002/advs.76951

**Published:** 2026-08-05

**Authors:** Jianjie Zhang, Lanhe Xu, Zhongzheng Zhang, Yongbo Li, Eric Li, Asoke K. Nandi, Bing Li

**Affiliations:** ^1^ School of Aeronautics Northwestern Polytechnical University Xi'an Shaanxi China; ^2^ School of Computing Engineering & Digital Technologies Teesside University Middlesbrough UK; ^3^ School of Mechanical Engineering Xi'an Jiaotong University Xi'an China; ^4^ Department of Electronic and Electrical Engineering Brunel University of London Uxbridge UK; ^5^ National Key Laboratory of Strength and Structural Integrity Xi'an China

**Keywords:** anisotropic wave propagation, composite materials, disordered metasurface, intelligent sensing, single‐sensor vibration sensing

## Abstract

Efficient vibration sensing in composite structures is often hindered by the anisotropic nature of wave propagation, which traditionally necessitates dense sensors and complex acquisition hardware. Compressed sensing, which relies on a predefined dictionary for sparse signal reconstruction, has emerged as a powerful tool to minimize sensor requirements; however, its integration into anisotropic media remains underdeveloped. Here we introduce a compact, single‐sensor vibration sensing (SSVS) system enabled by a disordered elastic metasurface that achieves high‐fidelity localization and identification of multi‐source vibrations in anisotropic composites. We show that the designed coupling between the disordered metasurface and anisotropic guided waves facilitates random phase modulation, which significantly reduces the coherence of the sensing matrix and overcomes the limitations of directional wave propagation in composites. Comprehensive numerical and experimental results verify that the proposed SSVS framework achieves high spatial resolution and robust performance regardless of diverse anisotropic lay‐ups, complex vibration conditions, or large curvature. Given its ease of fabrication and potential of embodied intelligence, this approach offers a new avenue for intelligent sensing in anisotropic mediums, offering broad potential for non‐destructive testing and structural health monitoring in advanced material systems.

## Graphical abstract

This work develops a single‐sensor vibration sensing (SSVS) framework that integrates disordered metasurfaces with compressive sensing. Comprehensive numerical and experimental validations demonstrate that, by manipulating anisotropic guided wave propagation through metasurfaces, the proposed SSVS framework enables high‐fidelity multi‐source vibration localization and signal identification with a single sensor across diverse anisotropic lay‐ups, complex vibration conditions, and curved structures with different curvatures.

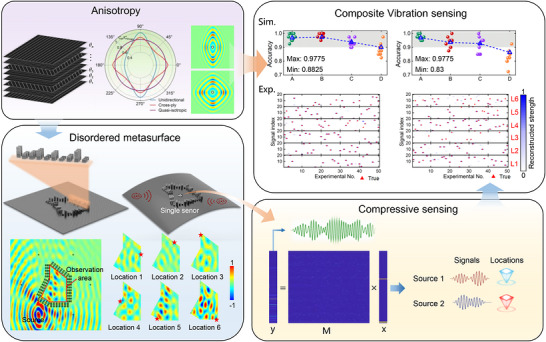

## 1 Introduction

Fibre‐reinforced composite materials have become indispensable in modern engineering owing to their exceptional specific strength/stiffness, lightweight nature, and fatigue resistance [1]. Through the service life of these advanced structures, vibration is an unavoidable and pervasive factor; thus, high‐fidelity vibration sensing is fundamental to intelligent sensing [2, 3, 4], structural health monitoring [5, 6, 7], and early‐warning systems [8, 9, 10]. However, the inherent anisotropy of laminated composites poses a formidable challenge. The strong directional dependence of elastic wave propagation leads to complex wavefront distortions and nonuniform measurement responses, which severely impede the efficiency and robustness of conventional vibration sensing frameworks.

To date, various vibration sensing paradigms have been developed, including vibration transmission path analysis [11, 12], array signal processing techniques [13, 14], and blind source separation methods [15, 16]. However, these methods typically rely on extensive multi‐sensor arrays and sophisticated data‐acquisition hardware, which inevitably increase the system complexity and power consumption. These limitations have motivated the development of more efficient sensing strategies that aim to reduce hardware requirements while preserving reconstruction performance. This trend is also evident in emerging sensing modalities such as terahertz (THz) sensing and imaging, where wave manipulation and information encoding are increasingly exploited to enhance sensing efficiency under hardware constraints [17, 18, 19]. In this context, compressed sensing (CS) has emerged as a transformative framework that exploits signal sparsity within a predefined dictionary to reconstruct high‐dimensional data from minimal measurements. Notably, the synergy between CS and advanced artificial materials, known as metamaterial compressed sensing (MCS), has redefined the boundaries of single‐sensor perception. Over the past decade, MCS has achieved remarkable success across electromagnetics, acoustics, and elastodynamics [20, 21, 22, 23, 24, 25], enabling breakthroughs like single‐pixel cameras [26, 27], single‐sensor sound source identification [28, 29, 30], and single‐sensor vibration localization [31, 32]. Nevertheless, existing MCS technologies remain largely confined to isotropic media. Furthermore, most reported implementations rely on multi‐layered 3D metamaterial architectures, which often result in bulky sensing systems and limit their integration into streamlined lightweight composite structures.

As a two‐dimensional alternative, elastic metasurfaces offer unprecedented control over the phase [33, 34, 35, 36, 37, 38], amplitude [39, 40, 41, 42], and trajectory [43, 44, 45, 46, 47, 48] of elastic waves within a subwavelength, compact footprint. The integration of metasurfaces with compressive sensing technology has further promoted the development of vibration sensing systems toward streamlined design. Li et al. [49] proposed a smart metasurface shaft (SMST) based on local resonance unit cells, achieving single‐sensor vibration identification for shaft structures. Zhang et al. [50] constructed a disordered coding metasurface using jagged unit cells, enabling vibration sensing for aluminum plate structures. In the aforementioned research, the introduction of metasurfaces overcame the bulky characteristic of MCS, thereby promoting a more compact and efficient sensing architecture. However, the sensing media considered in these works still are mainly limited to isotropic materials. In anisotropic composites, guided wave propagation strongly depends on both propagation direction and laminate stacking sequence, fundamentally altering the wavefield encoding process underlying compressive sensing and rendering metasurface design strategies developed for isotropic media no longer directly applicable. Up to now, no research has been reported on single‐sensor vibration sensing in composite structures to overcome anisotropic wave propagation and achieve reliable multi‐source signal reconstruction. Overcoming this requires a departure from deterministic periodicity. Disordered metasurfaces, characterized by complex multipath scattering and random phase modulation [51, 52, 53], provide a powerful physical mechanism to generate highly spatial uncorrelation. However, tailoring such stochastic interactions to break the rigid directional dependence of anisotropic propagation in composites and enhancing the spatio‐temporal incoherence of vibration signal for single‐sensor information recovery have, until now, remained a fundamental challenge.

**FIGURE 1:**
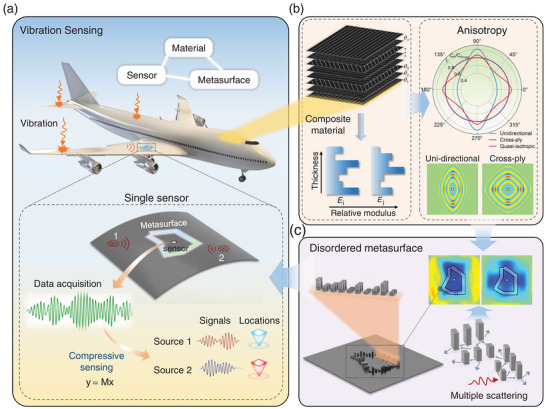
Schematic of single‐sensor vibration sensing in composite structures. (a) Concept of single‐sensor vibration sensing enabled by a disordered metasurface and compressed sensing for vibration source localization and identification. (b) Anisotropic characteristics of composite structures: variation of directional modulus with different stacking configurations (left), and polar plots of normalized phase velocity together with displacement field distributions under point source excitation for several representative laminates (right). (c) Schematic of the disordered metasurface design and the corresponding displacement field distribution of anisotropic guided waves under multiple scattering effects.

Here, we propose an SSVS system that integrates disordered metasurfaces with CS algorithms to achieve high‐precision, dictionary‐based multi‐source vibration localization and identification in composite structures (a schematic illustration is shown in Figure 1a). Through a synergistic coupling strategy between disordered metasurfaces and anisotropic guided waves, the correlation of the sensing system is effectively reduced to below 0.3, while simultaneously attenuating the direction dependency of guided wave propagation in composite materials. Numerical and experimental validations demonstrate that the SSVS framework enables high‐fidelity localization and accurate signal identification across diverse laminate configurations, maintaining exceptional robustness under complicated vibration conditions and within complex curved geometries. Characterized by its structural compactness and ease of fabrication, this framework establishes provides a promising approach for intelligent sensing in anisotropic media, offering transformative potential for wave manipulation, structural diagnostics, and the development of multifunctional advanced material systems.

## 2 Results and Discussion

### 2.1 Disordered Metasurface Design in Composite Medium

Composite laminates consist of individual layers stacked at various orientations, and differences in material stiffness between layers result in strong anisotropy in guided wave propagation. Based on classical laminate theory [54, 55], the equivalent material characteristics of these laminates can be determined (see Note S1). In this work, we implemented metasurface designs on composite laminates with three representative stacking sequences: unidirectional ([90]_8_), cross‐ply ([0/90]_4_), and quasi‐isotropic ([0/90/ − 45/45]_s_). Because the stacking configuration dictates the phase velocity and dispersion of guided waves, the wavelength varies substantially across different propagation directions and stacking angles, as shown in Figure 1b. To mitigate this inherent directional dependence, we propose a disordered metasurface design. Aligned with the dominant propagation directions of the anisotropic guided waves, the metasurface is arranged in an irregular hexagonal layout. In contrast to conventional periodic arrays, the spatial periodicity of the unit cells is deliberately disrupted to create a structure with long‐range disorder. This geometric disorder effectively enhances multiple scattering, thereby diversifying the propagation paths and intensifying modal coupling, as schematically illustrated in Figure 1c.

**FIGURE 2:**
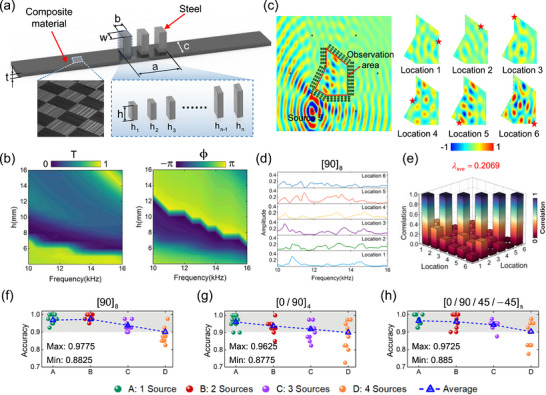
(a) Schematic of the metasurface unit cell design in composite medium. (b) Transmission (left) and phase variation (right) of the unit cell across varying pillar height and frequency ([90]_8_). (c) Displacement field distributions within the observation region at 13 kHz under vibration excitation from 6 different sources. (d) Numerically simulated FRFs of the 6 excitation sources. (e) Correlation distribution among the 6 vibration sources. (f–h) Sensing accuracy of the 3 representative stacking configurations under 4 excitation scenarios. In the plots, scatter points of different colors correspond to different numbers of excitation sources; each point represents the accuracy obtained from 50 tests, while the blue line with markers indicates the average accuracy over 10 rounds of tests for each excitation scenario.

To ensure that the unit cells operate in the subwavelength scale, the wavelength along the principal fibre direction of the unidirectional laminate was selected as the minimum reference value, which was then used to constrain the unit cell dimensions. As shown in Figure 2a, each unit cell consists of three subwavelength pillars made of steel (see Experimental Section for details), with dimensions *a* = 17 *mm*,  *b* = 3 *mm*,  *w* = 6 *mm*,  *c* = 12 *mm*,  *t* = 1.5 *mm*, and a height *h* designed within the range of 4–16 mm. The transmission and phase of the unit cell were analyzed along the principal fibre direction over a frequency range of 10–16 kHz, as shown in Figure 2b and Figure S1. As the height increases, the transmission coefficient of the unit cell shows a non‐monotonic variation, and the phase shift covers nearly the full 2π range. These characteristics demonstrate that, through appropriate tuning *h*, random modulation of both wavefront phase and amplitude can be achieved. Wavefield simulations were then performed by arranging unit cells with randomly assigned heights. For a given stacking configuration, the disordered metasurface induces strong multiple scattering effects, resulting in highly complex guided‐wave propagation compared with the cases without a metasurface or with a periodic array, as shown in Figure S2. The resulting displacement fields exhibit high spatial complexity, which significantly enhances the incoherence of signal transmission. This spatial incoherence is highly desired, as it facilitates the construction of an effective measurement matrix for compressed sensing.

Based on the unit‐cell analysis and the characteristics of disordered arrangements, a spatially disordered metasurface is constructed to tailor anisotropic guided waves in composite laminates. The distribution of the unit‐cell height *h_i_* is designed using Equation (1):

(1) $$h_{i}=M\left\{\sum_{n=1}^{3}\left(\alpha_{n}\cdot \frac{\bar{L}\left(\theta_{i}\right)}{{\bar{L}}_{max}}\right)+\zeta_{i}\right\}$$

where M denotes a mapping operator that constrains the result within the manufacturable height range. $\alpha_{n}\in \left[0,\;{\bar{L}}_{min}/4\right]$ is the weighting coefficient associated with the *n*‐th laminate configuration, and $\bar{L}\left(\theta_{i}\right)$ represents the equivalent wavelength along the *i*‐th propagation direction for different lay‐ups. ζ_*i*_ is a stochastic perturbation term following a probability distribution, introduced to generate height fluctuations and enhance the randomness among adjacent unit cells. In contrast to isotropic media, the inherent anisotropy of composite laminates induces direction‐dependent variations of effective mechanical parameters $\bar{E}\left(\theta_{i}\right),\;\bar{G}\left(\theta_{i}\right),\bar{\nu }\left(\theta_{i}\right)$, resulting in strongly directional guided wave propagation. The design of a disordered metasurface in composites must explicitly account for this anisotropic propagation behavior. The equivalent wavelength $\bar{L}\left(\theta_{i}\right)$ in Equation (1) varies with the propagation angle θ_*i*_and determines the characteristic design scale of the metasurface height *h_i_*. The wavelength and wavenumber satisfy $\bar{L}\left(\theta_{i}\right)=2\pi /\bar{k}\left(\theta_{i}\right)$, with $\bar{k}\left(\theta_{i}\right)\propto f\left(\theta \right)$, where $f_{\theta }=1/{\bar{E}}_{i}$ and ${\bar{E}}_{i}$ is the effective Young's modulus along the *i*‐th direction, which can be obtained from the transformed‐axis equation:

(2) $$f_{\theta }=\frac{\cos^{4}\theta_{i}}{{\bar{E}}_{1}}+\frac{\sin^{4}\theta_{i}}{{\bar{E}}_{2}}+\left(\frac{1}{{\bar{G}}_{12}}-\frac{2{\bar{\nu }}_{12}}{{\bar{E}}_{1}}\right)\sin^{2}\theta_{i}\cos^{2}\theta_{i}$$

where ${\bar{E}}_{1}$, ${\bar{E}}_{2}$, ${\bar{G}}_{12}$, and ${\bar{\nu }}_{12}$ are the equivalent elastic constants of the laminate, and θ_*i*_ denotes the angle of the *i*‐th direction. The coefficients α_*n*_ (*n* = 1, 2, 3) serve as weighting factors for different laminate configurations ([90]_8_, [0/90]_4_, [0/90/ − 45/45]_*s*_) in Equation (1), allowing control over the relative contribution of each laminate and thereby optimizing guided wave scattering and coupling across layers. Ultimately, the strong coupling between the disordered metasurface and anisotropic guided waves induces multiple scattering and local impedance mismatch, enhancing the incoherence of spatial vibration responses and promoting a more balanced directional energy distribution.

To validate the modulation capability of the disordered metasurface, six excitation sources at various orientations were applied to the composite laminate. Numerical simulations of the displacement field in a unidirectional laminate were performed at 13 kHz, as shown in Figure 2c. The right panel presents a comparison of displacement fields within the observation region under excitations from different directions, with the central measurement point's amplitude variation expressed as *η* = 20*log*(*w_i_*/*w_min_*) for the cases with and without the metasurface (detailed in Note S2 and Table S3). The results demonstrate that the coupling between the disordered metasurface and anisotropic guided waves generates complex and highly distinct propagation patterns for all excitation directions. These findings indicate that the designed disordered metasurface can effectively disrupt the original directional propagation behavior, enabling wavefield responses with richer and more diverse information content across different directions.

To evaluate the vibration transmission from each source, the frequency response function (FRF) is defined as $W_{i}\left(f_{\theta }\right)=w_{out}^{i}/w_{in}^{i}$, where $w_{\mathrm{out}}^{i}$ and $w_{\mathrm{in}}^{i}$ represent the fast Fourier transform (FFT) of the output signal at the center of the observation region and the input signal of the *i*‐th excitation source, respectively. The FRF curves for each source were obtained through numerical simulations, as shown in Figure 2d and Figure S3. The cross‐correlation coefficient λ between any two sources is then calculated according to Equations (3 and 4):

(3) $$\begin{array}{ccc} & & \lambda \left(W_{i}\left(f_{\theta }\right),W_{j}\left(f_{\theta }\right)\right)\\ & & \;=\frac{\sum_{k}\left[W_{i}\left(\omega_{k},f_{\theta }\right)-{\bar{W}}_{i}\left(f_{\theta }\right)\right]\left[W_{j}\left(\omega_{k},f_{\theta }\right)-{\bar{W}}_{j}\left(f_{\theta }\right)\right]}{\sqrt{\sum_{k}{\left(W_{i}\left(\omega_{k},f_{\theta }\right)-{\bar{W}}_{i}\left(f_{\theta }\right)\right)}^{2}}\sqrt{\sum_{k}{\left(W_{j}\left(\omega_{k},f_{\theta }\right)-{\bar{W}}_{j}\left(f_{\theta }\right)\right)}^{2}}}\;\;\;\; \end{array}$$

(4) $$\lambda_{ave}=\frac{1}{m\left(m-1\right)}\;\sum_{i\neq j}\left|\lambda \left(W_{i}\left(f_{\theta }\right),W_{j}\left(f_{\theta }\right)\right)\right|$$

in these expressions, *k* denotes the frequency index, *W_i_* (*f*_θ_) and *W_j_* (*f*_θ_) represent the mean values of the FRFs, *m* is the number of excitation sources, and λ_*ave*_ is the average cross‐correlation coefficient across all source pairs, ranging from 0 to 1, with values closer to 0 indicating stronger mutual incoherence. In this work, the SSVS system is considered to exhibit satisfactory incoherence when λ_*ave*_ < 0.3. Figure 2e and Figure S3 show the distribution of cross‐correlation coefficients among multiple vibration sources for the three different stacking configurations within the same disordered metasurface system, with λ_*ave*_ values of 0.2069, 0.2235, and 0.1794, respectively. The reduced correlation originates from the combined effects of local resonances, multiple scattering, and mode conversion induced by the disordered metasurface. These wave phenomena alter the propagation and energy distribution of guided waves, leading to more diverse wavefield responses under different excitation sources. Consequently, the intrinsic direction‐dependent characteristics associated with material anisotropy are effectively weakened, resulting in reduced correlations among responses generated by different vibration sources. A quantitative comparison of the correlation characteristics for the without metasurface and representative metasurface designs (periodic, quasi‐periodic, and random) is presented in Note S3 and Figure S9 to evaluate the influence of structural disorder on sensing performance. The simulation results indicate that the designed disordered metasurface is compatible with multiple composite laminate configurations while maintaining highly uncorrelated transmission characteristics, providing suitable conditions for constructing an ideal measurement matrix consistent with compressed sensing theory.

### 2.2 Vibration Sensing in Composite Laminates

#### 2.2.1 SSVS Strategy in Anisotropic Medium

Building on the enhanced mutual incoherence of anisotropic vibration transmission introduced by the disordered metasurface, this work implements a compressed sensing (CS) based approach for single‐sensor composite structure vibration detection. Within the compressed sensing framework, the vibration sensing model can be expressed as *y* = *Mx*, where *y* is the data vector measured by the single sensor, *x* is the target vector to be reconstructed, and $M_{l\times k}=\left[\begin{array}{ccc} M_{11} & \cdots & M_{1\times k}\\ \vdots & \ddots & \vdots \\ M_{l\times 1} & \cdots & M_{l\times k} \end{array}\right]$ is the measurement matrix including prior knowledge. This matrix is determined by the system's FRFs in combination with the excitation signals, representing both the spatial locations of the sources and the corresponding signal content, as expressed in Equation (5):

(5) $$M_{l}\;\left(\omega_{k}\right)=W_{m}\;\left(\omega_{k}\right)\cdot S_{n}\;\left(\omega_{k}\right)=\Phi \left(\omega_{k}\right)\Psi_{m}\left(\omega_{k},f_{\theta }\right)\cdot S_{n}\left(\omega_{k}\right)$$

in these expressions, *m* and *n* denote the numbers of excitation sources and signals, respectively, and *l* = *m* × *n* represents the number of columns in the measurement matrix. *W_m_* (ω_*k*_) corresponds to the FRFs of the sensing system, which is jointly determined by the anisotropic propagation operator Ψ_*m*_(ω_*k*_,*f*_θ_) and the modulation operator of the disordered metasurface Φ(ω_*k*_). *S_n_* (ω_*k*_)represents the spectral amplitude of the excitation signal. According to compressed sensing theory, to ensure stable signal reconstruction, the measurement matrix is required to satisfy the Restricted Isometry Property (RIP) [56], as expressed in Equation (6):

(6) $$\left(1-\delta_{s}\right){∥x∥}_{2}^{2}\leq {∥Mx∥}_{2}^{2}\leq \left(1+\delta_{s}\right){∥x∥}_{2}^{2}$$

there, $\delta_{s}\in \left(0,1\right)$ denotes the restricted isometry constant. Due to the combinatorial complexity involved in the direct computation of the RIP constants, exact evaluation remains intractable for large‐scale practical systems. Existing studies have established that the mutual coherence between column vectors of the sensing matrix can serve as a computable surrogate upper bound for RIP characterization [57]. Theoretically, lower mutual coherence implies that the column vectors of the sensing matrix are closer to orthogonality, thereby increasing the probability of satisfying the RIP condition. Combined with the correlation analysis presented in Section 2.1, it can be observed that the proposed disordered metasurface significantly reduces the correlation among responses generated by different vibration sources, indicating a low coherence structure of the sensing matrix. Therefore, from a statistical perspective, the system is more likely to satisfy the RIP constraint, providing a theoretical foundation for stable compressive sensing reconstruction. This further demonstrates that the proposed SSVS system enables high‐precision reconstruction of anisotropic vibration signals.

The sensing procedure is implemented in two stages, localization and identification, for concurrent determination of the source locations and their excitation signals. Specifically, multiple positions on the composite laminates are simultaneously excited, with each excitation signal randomly selected from a library of 20 distinct signals (see Experimental Section for the construction of the testing signals). The center of the observation region is designated as the single sensor location to collect the vibration responses modulated by the disordered metasurface. The signals received at this single sensor are processed using the two‐step iterative shrinkage/thresholding (TWIST) algorithm [58], enabling reconstruction of the target vector *x*, which represents both the source positions and the signal indices. A detailed description of the sensing procedure is provided in Note S4 and Figure S10.

In the numerical simulations, excitation was applied at each source location to compute the FRFs, which were subsequently combined with the random signals to construct the measurement matrix *M*. Sensing tests were conducted under 1, 2, 3, and 4 simultaneous excitations, with each round randomly selecting 50 combinations of source positions and signals, and a total of 10 independent rounds were performed. The average accuracy across these rounds was used to evaluate sensing performance. In particularly, for multi‐source sensing, a successful identification requires both the correct localization of excitation sources and the accurate reconstruction of the corresponding signals associated with each source. As shown in Figure 2f–h, for the three different laminate stacking configurations, the average sensing accuracy exceeded 90% for 1, 2, and 3 simultaneous excitations, with 1 and 2 source tests consistently achieving over 90% accuracy in each round. Although the sensing task becomes more challenging as the number of excitation sources increases, the overall accuracy remains at a high level. Furthermore, to further verify the effectiveness of the proposed disordered design in improving sensing performance, we compared the sensing results under different metasurface designs. The corresponding results are presented in Note S5 and Figure S11.

These simulation results demonstrate that the proposed approach, combining the disordered metasurface with compressed sensing, can achieve average sensing accuracy above 90% in composite laminates using a single sensor. It should be noted that this approach is essentially a dictionary‐based method, and its reconstruction performance relies on the consistency between the calibrated responses and actual excitations. Therefore, deviations in source locations or excitation signals beyond the predefined conditions may introduce mismatch between the sensing matrix and the actual responses, leading to reduced reconstruction accuracy. Moreover, the conclusions from the numerical simulations were confirmed by experimental results, further validating the feasibility and practical applicability of the method.

#### 2.2.2 Experimental Validations

**FIGURE 3:**
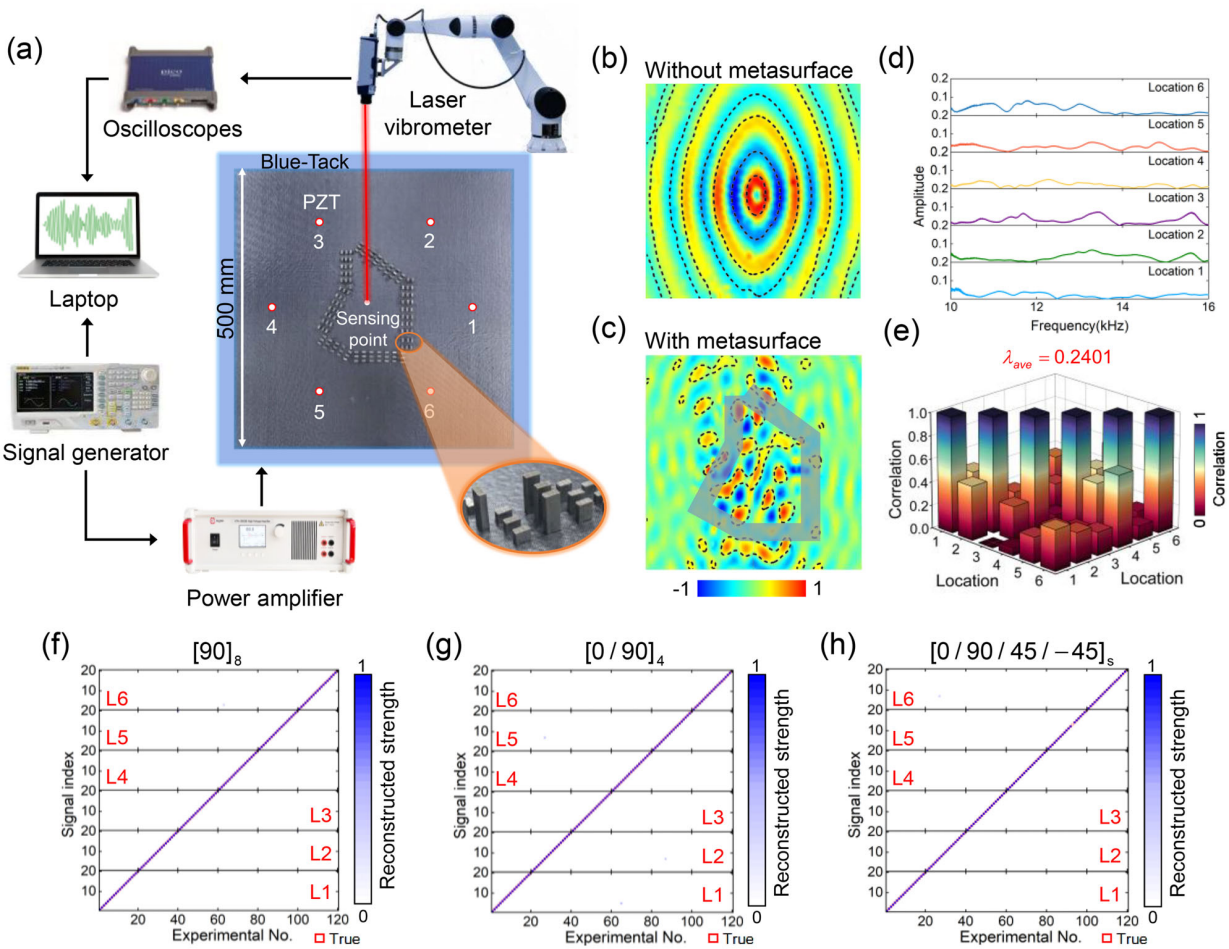
(a) Experimental procedure for the SSVS system. (b) Wavefield measurements over a 150 mm × 150 mm region at the center of the unidirectional laminate without the metasurface. (c) Wavefield measurements over a 270 mm × 270 mm region with the disordered metasurface introduced. (d) and (e) Experimentally measured FRFs of 6 excitation sources and the correlation distribution among the sources. (f–h) Single‐source sensing results for three laminate stacking configurations. Blue regions indicate reconstructed strength, and red boxes mark the actual excitation positions and signals.

To validate the effectiveness of the proposed SSVS system and the accuracy of the numerical simulations, experiments were conducted on three types of laminated plates, following the procedure illustrated in Figure 3a. The laminates measured 500 mm × 500 mm × 1.5 mm, with detailed experimental specifications provided in the Experimental Section. Initially, wavefield measurements were performed without the disordered metasurface. An excitation was applied at the center of the laminate, and the transient displacement field is shown in Figure 3b and Figure S4. In the unidirectional laminate, guided waves propagated predominantly along the 90° direction, consistent with the simulation results. Subsequently, wavefield measurements were performed with the disordered metasurface system installed. As shown in Figure S4, the guided waves largely retained their original propagation characteristics before 0.5 ms, but after interacting with the disordered metasurface, their propagation behavior changed significantly. Figure 3c shows the wavefield at 1.5 ms, where the originally distinct directional propagation pattern is completely disrupted.

To evaluate the correlation in the experiments, frequency sweep excitations were applied at 6 different positions over a range of 10 kHz to 16 kHz. As shown in Figure 3d,e and Figure S4, the correlation coefficients between any two vibration sources are distributed within the range of 0.001–0.6. The results indicate that the average correlation coefficients λ_ave_ among the sources are 0.2401 ([90]_8_), 0.2027 ([0/90]_4_), and 0.2329 ([0/90/ − 45/45]_*s*_), respectively. These experimental results clearly demonstrate that the proposed disordered metasurface system effectively disrupts the directional dependence of vibration propagation in composite laminates and enhances the complexity and randomness of the transmitted waves, thereby establishing a highly incoherent vibration sensing system. This low correlation transmission characteristic provides a critical foundation for subsequent compressed sensing‐based vibration localization and identification.

In the vibration sensing experiments, 20 random excitation signals were sequentially excited at 6 source locations, resulting in a total of 120 experimental trials. The output response measured at a single sensing point was recorded and transformed into the frequency domain using the FFT, thereby constructing the observation matrix *M*. In practice, the experimentally acquired matrix *M* contains redundant information and measurement noise; therefore, principal component analysis (PCA) was employed as a preprocessing step (see Note S6 and Tables S4 and S5 for details and parameter selection).

During the sensing process, a correct identification is defined by a target component with a reconstruction strength exceeding 0.5 (see Note S7 and Table S6 for the threshold selection), as well as the accurate recognition of both the source location and its corresponding excitation signal. The results of single source sensing are shown in Figure 3f–h, where the red boxes indicate the true excitation positions and signals, and the blue regions represent the reconstructed results obtained via compressed sensing, with darker colors corresponding to higher reconstruction strength. As observed, the reconstructed results exhibit excellent agreement with the actual excitation conditions. Based on the above identification criterion, single‐source sensing on all three laminate configurations achieved an accuracy of 100% within the same disordered metasurface system, maintaining a consistently high level comparable to the numerical results, which exceeded 97%. Such high precision demonstrated in single‐source excitation within composite laminates provides a strong foundation for subsequent multi‐source vibration sensing experiments.

**FIGURE 4:**
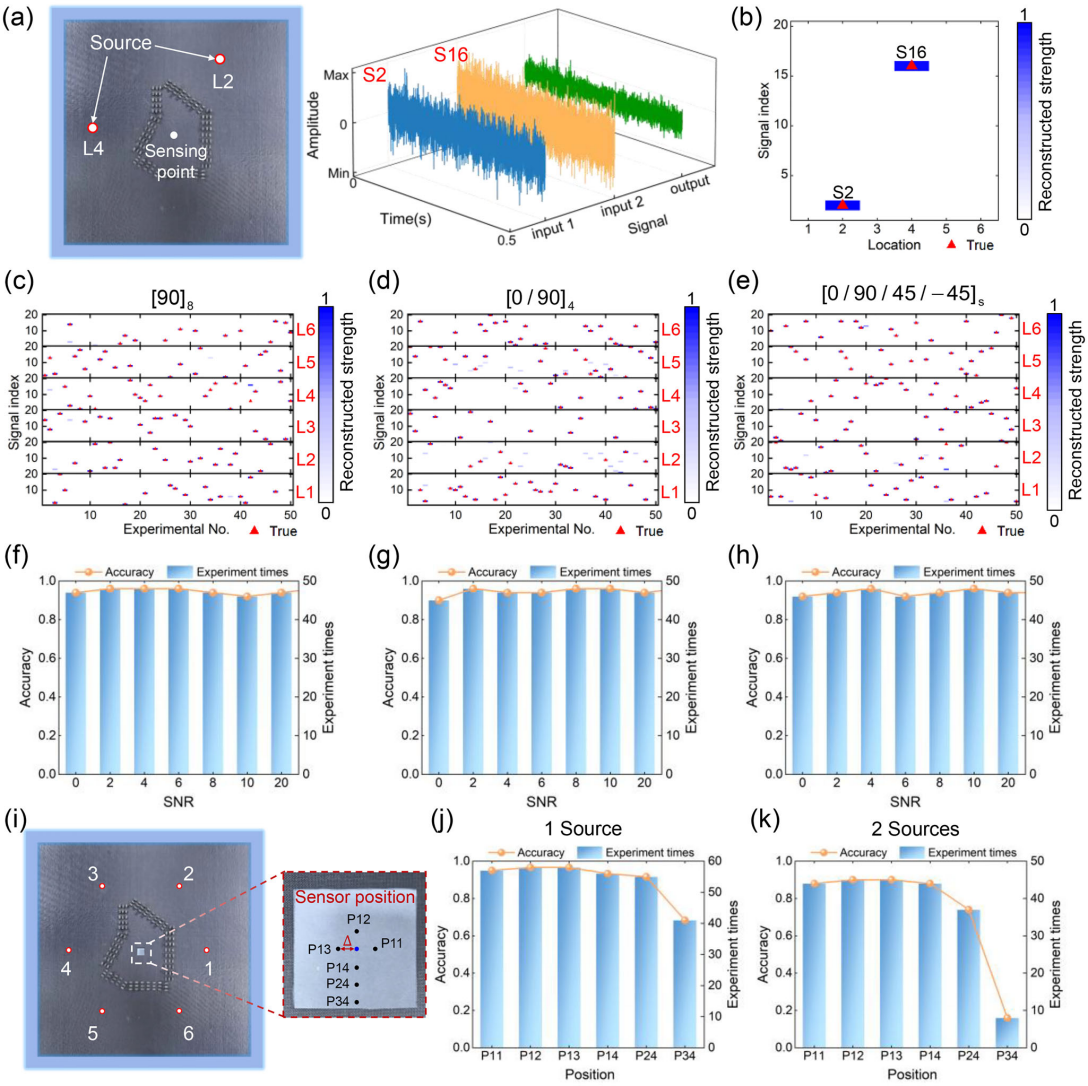
(a) Schematic of the double‐source sensing experiments. (b) Reconstructed results corresponding to (a), where the blue regions indicate the reconstructed strength and the red triangles mark the actual excitation positions and signals. (c–e) Results of 50 experimental trials for the three laminate configurations, with achieved accuracies of 96% (c), 96% (d), and 94% (e), respectively. (f–h) Number of correctly identified cases and sensing accuracy under different SNRs. (i) Schematic of the robustness validation experiment for different sensor positions. (j–k) Sensing accuracy of 1‐source and 2‐source cases at different sensor positions.

In the double‐source excitation experiments, as illustrated in Figure 4a, two different random excitation signals were simultaneously excited at two different locations, with the reconstructed results shown in Figure 4b. In the Figure, the blue regions denote the reconstructed results, while the red triangles indicate the true source locations and their corresponding signal types. For each laminate configuration, 50 double‐source sensing experiments were conducted, and the experimental results are shown in Figure 4c–e. The achieved sensing accuracies were 96% ([90]_8_), 96% ([0/90]_4_), and 94% ([0/90/ − 45/45]_*s*_), respectively. Among all 150 experiments, only 7 cases failed to be correctly identified, and in five cases, only one of the four required pieces of information was misidentified. Representative reconstruction details for the 13th, 28th, and 40th trials are provided in Figure S5.

To evaluate the robustness of the SSVS system, Gaussian white noise with different SNRs was first introduced in the double source sensing experiments (SNR = 0, 2, 4, 6, 8, 10, and 20 dB), with the results shown in Figure 4f–h. While the presence of noise slightly reduced the sensing accuracy, the overall performance remained above 90%. Representative reconstruction details under selected noise levels (SNR = 0, 4, and 8 dB) are presented in Figure S6. Furthermore, to investigate the influence of sensor position deviation on sensing performance, robustness experiments with different sensing locations were conducted in the cross‐ply laminate. As illustrated in Figure 4i, the central position (blue point) was selected as the reference sensing position to construct the measurement matrix *M*, and 6 additional sensing positions with offsets were arranged around it for testing, with a spacing of Δ = 2 *mm* between adjacent sensing points. The results shown in Figure 4j,k indicate that when the sensor is located at positions with the same distance from the reference point (P11, P12, P13, and P14), the corresponding sensing accuracies remain nearly identical. As the deviation distance from the central sensing position increases, the sensing performance gradually decreases. For single source localization, the sensing accuracy remains above 90% when the offset distance satisfies Δ ≤ 4 *mm*. For double source localization, the accuracy remains above 88% when Δ ≤ 2 *mm*, while it decreases to 74% at Δ = 4 *mm*. Signal reconstruction results of double sources sensing at several sensing positions are presented in Figure S7. These results demonstrate that sensor position deviations within a certain range do not significantly affect the sensing capability of the system.

In addition to the above experimental validations, further analyses were conducted on the cross‐ply laminate to investigate potential disturbance factors that may arise in practical engineering applications, including excitation source position deviation (Note S8 and Figure S12), temperature variations (Note S9 and Figure S13), metasurface bonding conditions (Note S10 and Figure S14), and boundary condition changes (Note S11 and Figure S15). The corresponding results indicate that, within the limited disturbance ranges investigated, the proposed SSVS system maintains an acceptable sensing performance under various environmental perturbations and structural variations.

These experimental results indicate that the proposed SSVS system is capable of separating mixed excitation signals in composite materials and performing vibration localization and identification within the predefined sensing dictionary under multiple excitation conditions. The system maintains relatively stable sensing performance under noise interference and moderate environmental perturbations within the investigated range. However, when environmental variations exceed the calibrated conditions, the sensing matrix may require recalibration to compensate for the mismatch between the measured responses and the actual structural states. This work provides a feasible approach for single‐sensor vibration sensing in complex composite structures.

### 2.3 SSVS in Composite Shell Structures

**FIGURE 5:**
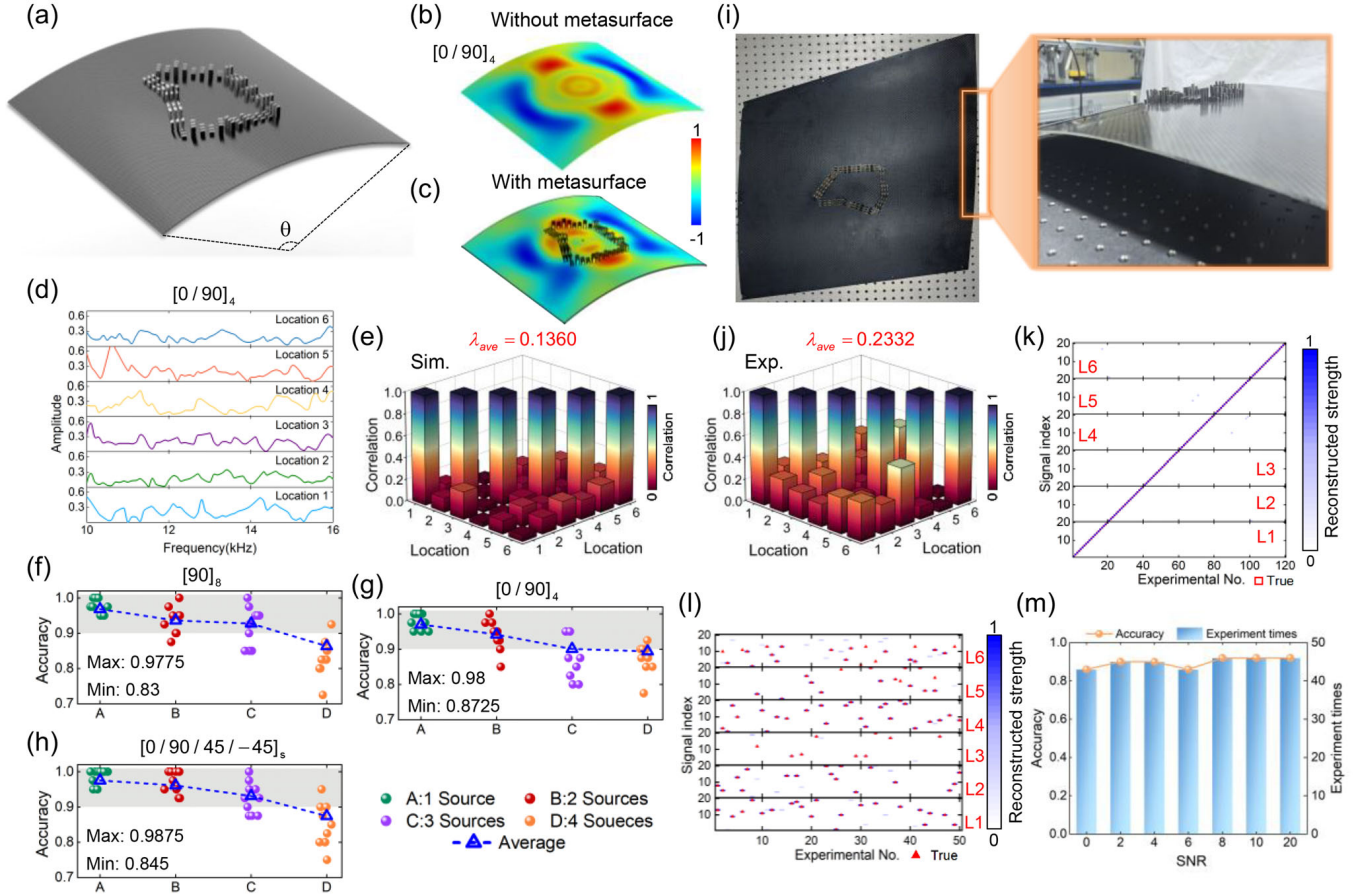
(a) Schematic of the composite shell structure integrated with the disordered metasurface, with a central angle of θ = 45°. (b) Displacement field distribution in the orthotropic composite shell without a metasurface. (c) Displacement field distribution in the cross‐ply composite shell with the disordered metasurface. (d), (e) Simulated FRFs of the 6 excitation sources and the corresponding source correlation coefficient distributions. (f–h) Sensing accuracy of the three representative laminate shell configurations under four excitation scenarios. (i) Composite shell specimen used in the experiments. (j) Correlation coefficient distribution among responses from different excitation sources in the composite shell structure. (k) Results of single‐source sensing experiments. (l) Results of dual‐source sensing experiments. (m) Number of correctly identified cases and sensing accuracy of dual‐point sensing experiments under different SNR conditions.

To explore the applicability of the SSVS system in more complex structures, this work extends its focus to composite shell structures. Compared to plates, shell structures introduce geometric curvature, which simultaneously affects wave propagation due to both surface effects and material anisotropy. This presents a higher challenge for wave control and sensing. Therefore, conducting vibration sensing research on composite shells is crucial for systematically assessing the robustness and potential applicability of the sensing system in practical structural environments. As shown in Figure 5a, the shell structure model has a central angle of θ = 45°, with both the arc length and width measuring 400 mm. Without the metasurface, the wave propagation in the composite shell ([0/90]_4_) is depicted in Figure 5b. Due to the curvature, the wave propagation differs from that in plates but still retains the orthogonal anisotropic propagation characteristics.

When the same disordered metasurface configuration used in Section 2.2 was applied to the shell surface, the anisotropic propagation of guided waves was similarly suppressed, as shown in Figure 5c. This demonstrates that the designed disordered metasurface remains effective in manipulating guided waves within composite shell structures and enhances the transmission decorrelation among multiple vibration sources. Using Equations (1 and 2), the correlation coefficients between any two vibration sources are found to be within 0.02–0.48, indicating low inter‐source correlation. The average correlation coefficients λ_*ave*_ among the 6 sources were calculated to be 0.1819 ([90]_8_), 0.1360 ([0/90]_4_), and 0.1777 ([0/90/ − 45/45]_*s*_). The corresponding frequency response functions and the distributions of inter source correlation coefficients are presented in Figure 5d,e and Figure S8. These results provide strong support for subsequent high‐precision vibration sensing in composite shell structures.

In composite shell structures, vibration sensing tests were conducted for excitation scenarios involving 1, 2, 3, and 4 sources. For each excitation condition, 10 independent trials were performed, and the averaged results were used to evaluate the sensing accuracy. The corresponding sensing outcomes are presented in Figure 5f–h. For all three laminate configurations, the average sensing accuracy exceeded 90% under single‐source and two‐source excitations, demonstrating sensing performance comparable to that observed in plate structures. Furthermore, when the number of excitation sources increased to three, the sensing accuracy for both the unidirectional and quasi‐isotropic laminates remained above 90%. As the number of excitation sources increased further, a gradual reduction in sensing accuracy was observed; nevertheless, the overall accuracy consistently remained above 80%. Furthermore, to further investigate the influence of curvature variations on vibration sensing performance, numerical simulations under different curvature conditions were conducted for the cross‐ply laminate, and the corresponding results are presented in Note S12 and Figure S16. The simulation results demonstrate that, although vibration propagation behavior in shell structures becomes more complex due to curvature effects, the proposed multi‐source vibration sensing system for composite materials still exhibits promising applicability.

To validate the sensing capability of the proposed SSVS system in curved composite structures, vibration sensing experiments were conducted on a composite shell structure. As shown in Figure 5i, a composite shell specimen with a certain curvature was fabricated, and the disordered metasurface was integrated onto its surface. During the experiments, the response correlations among 6 predefined excitation source locations were measured, with an average correlation coefficient of λ_*ave*_ =  0.2332. The correlation coefficient distribution among different excitation source responses is presented in Figure 5j. The sensing results are shown in Figure 5k–l. Under single‐source excitation conditions, the SSVS system achieved 100% sensing accuracy, while under double‐source excitation conditions, it still reached a sensing accuracy of 92%. Furthermore, to evaluate the sensing capability of the system under noisy conditions, double‐source sensing experiments were further conducted under different SNR conditions. The results demonstrate that the sensing accuracy remains above 86%, indicating certain noise resistance of the proposed system.

The above experimental results validate the feasibility of the proposed SSVS system for curved composite structures, demonstrating that the method can achieve effective localization of predefined vibration sources and accurate identification of the corresponding signal types under complex structural response conditions. These results provide experimental support for the further application of the proposed approach in vibration sensing of curved composite structures.

## 3 Conclusions

In conclusion, we have established a new SSVS framework for vibration sensing in highly anisotropic composite materials by synergizing disordered metasurfaces with compressed sensing theory. By utilizing purposefully designed geometric disorder as a physical encoding layer, our system actively disrupts inherent anisotropic wave symmetries via complex subwavelength multipath scattering. This wave‐matter interaction deterministically transforms structurally constrained, directionally biased guided waves into a highly spatially incoherent measurement basis, perfectly satisfying the rigorous mathematical prerequisites for single‐sensor signal recovery. Consequently, this framework demonstrates effective sensing performance, achieving high‐fidelity identification of multi‐source vibrations within predefined candidate sets using only a single sensor across diverse composite architectures, regardless of extreme anisotropy, large geometry curvature or complex vibration conditions. By conceptually shifting the burden of signal processing from the digital domain to the physical realm, this methodology obviates the reliance on dense, high‐channel‐count sensor networks. Ultimately, this principle of wave‐based physical encoding extends far beyond conventional vibration monitoring, providing a versatile platform that lays the groundwork for the next generation of intelligent metamaterials, non‐destructive evaluation, and autonomous diagnostics in complex continuous media.

## 4 Experimental Section

### 4.1 Numerical Simulations

All numerical simulations in this work were performed using COMSOL Multiphysics 6.2, employing the Solid Mechanics physics interface. The FRFs were computed in the frequency domain. The simulation model is shown in Figure 2c, with a computational domain of 400 mm × 400 mm. Perfectly matched layers (PMLs) with dimensions of 50 mm × 500 mm were applied along the outer boundaries to eliminate the effects of boundary reflections. The disordered metasurface was fabricated from 304 stainless steel, with material properties including Young's modulus *E* = 200 *GPa*, Poisson's ratio *μ* = 0.3, and density *ρ* = 7930 *kg*/*m* ^3^. The composite laminate was based on T700 epoxy prepreg, and the corresponding equivalent material properties are provided in Note S1 and Tables S1 and S2.

### 4.2 Experimental Measurement Setup

The composite laminate specimens used in the experiments were fabricated using an autoclave molding (ACM), while the metasurface pillars were manufactured by wire electrical discharge machining, achieving a dimensional tolerance of 0.2 mm. Images of the experimental specimens are shown in Figure 3a. The metasurface pillars were bonded onto the laminate surface, and blue tack was applied along the laminate boundaries to absorb reflected waves. Considering that the additional mass introduced by the steel pillar metasurface may alter the dynamic response of the composite structure, the influence of the metasurface on the structural dynamic characteristics was further analyzed, and the corresponding results have been provided in Note S13 and Table S7 and Figure S17. The experimental procedure is as follows. Excitation signals were generated by a signal generator (RIGOL DG4062) and amplified using a power amplifier (ATA 2022B) before being applied to piezoelectric patches. The resulting vibration responses were captured at the measurement region or selected sensing point using a noncontact laser vibrometer (Polytec VFX‐1‐110) and simultaneously transmitted to a laptop computer via an oscilloscope (PicoScope 4000 Series).

During wavefield measurements, a reflective film was applied to the back surface of the laminate (the side without the metasurface) to define the measurement region and prevent interference from reflections off the metasurface. A piezoelectric patch was bonded at the center of the laminate, and a pulse signal with a central frequency of 13 kHz was generated by the signal generator, as expressed in Equation (7):

(7) $$w\;\left(t\right)=A\left[1-\cos\left(\frac{2\pi ft}{10}\right)\sin\left(2\pi ft\right)\right]$$

During data acquisition, the laser vibrometer system controlled a robotic arm to move the sensor tip across the measurement region, recording the vibration velocity point by point and transmitting the data from each measurement point to the computer.

During the sensing tests, the laser vibrometer collected vibration data only at the center of the laminate. Piezoelectric patches were attached around the circumference of the metasurface, with adjacent patches spaced at 60° and located 150 mm from the measurement point. Random excitation signals were generated in MATLAB, as expressed in Equation (8):

(8) $$S_{n}\;\left(t\right)=\sum_{i=1}^{20}A_{i}\sin\left(2\pi f_{i}t+\varphi_{i}\right)$$

here, t∈[0,1], $A_{i}\in \left[0,1\right]$, $f_{i}\in \left[10,16\right]$ kHz, $\varphi_{i}\in \left[0,2\pi \right]$. Each signal was normalized to ensure equal energy. The prepared signals were then loaded into the signal generator and applied sequentially as excitations. Each excitation was maintained for 0.5 s, with a 1 s pause between consecutive excitations, and the response signals were acquired at a sampling rate of 500 kHz.

## Author Contributions

**Jianjie Zhang**: writing – original draft, methodology, software, visualization, formal analysis, validation, data curation, investigation. **Lanhe Xu**: methodology, software, validation, investigation. **Zhongzheng Zhang**: methodology, software, validation, investigation. **Yongbo Li**: methodology, validation. **Eric Li**: writing – review and editing, methodology, investigation. **Asoke K. Nandi**: methodology, investigation, writing – review and editing. **Bing Li**: conceptualization, funding acquisition, writing – review and editing, supervision, resources, investigation, methodology, project administration.

## Conflicts of Interest

The authors declare no conflicts of interest.

## Supporting information




**Supporting File**: advs76951‐sup‐0001‐SuppMat.pdf.

## Data Availability

All the data and original code in the paper or the supplemental information are available from the corresponding authors upon reasonable request.
