# Supplementary material for: Single‐Sensor Vibration Sensing in Anisotropic Composites Enabled by Disordered Metasurfaces
Source: Adv Sci (Weinh). 2026 Aug 5;13(60):e76951. doi: 10.1002/advs.76951 (PMC13440195; doi:10.1002/advs.76951)
Supplement: Supplementary file 1 [file ADVS-13-e76951-s001.pdf]

Supporting Information

Single-sensor vibration sensing in anisotropic composites enabled by disordered metasurfaces

Jianjie Zhang[1], Lanhe Xu[1], Zhongzheng Zhang[1], Yongbo Li[1], Eric Li[2], Asoke K. Nandi[3, 4], Bing Li[1, 5, *]

[1] School of Aeronautics, Northwestern Polytechnical University, Xi'an, Shaanxi, 710072, China.

[2] School of Computing, Engineering & Digital Technologies, Teesside University, Middlesbrough, UK

[3] School of Mechanical Engineering, Xi'an Jiaotong University, Xi'an, China

[4] Department of Electronic and Electrical Engineering, Brunel University of London, Uxbridge, UB8 3PH, United Kingdom

[5] National Key Laboratory of Strength and Structural Integrity, Xi'an, China

***Corresponding author E-mail address**: bingli@nwpu.edu.cn

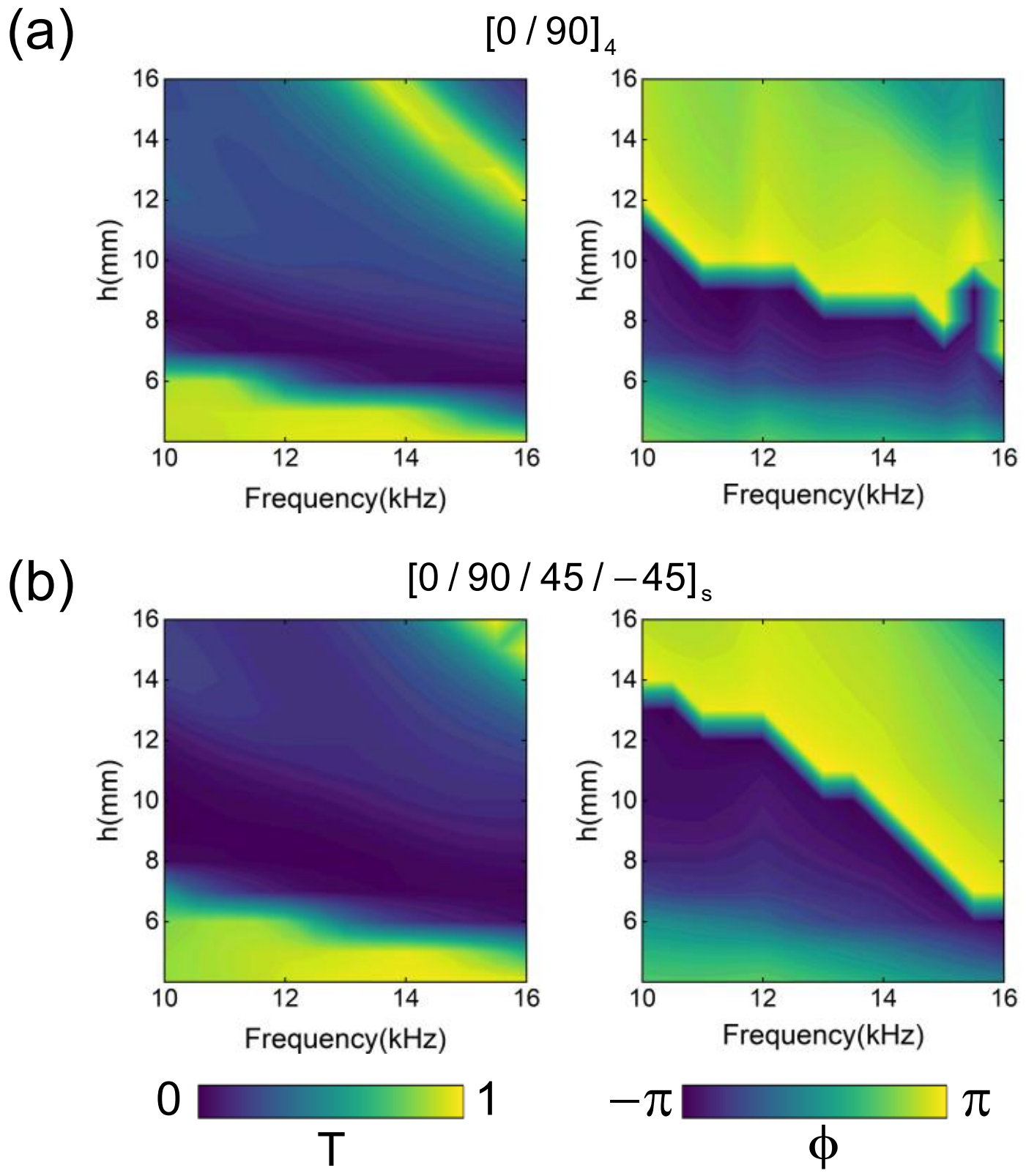

Figure S1. Analysis of the transmission amplitude and phase of disordered metasurface unit cells on cross-ply (a) and quasi-isotropic (b) laminates with respect to pillar height (4-16 mm) and frequency (10-16 kHz).

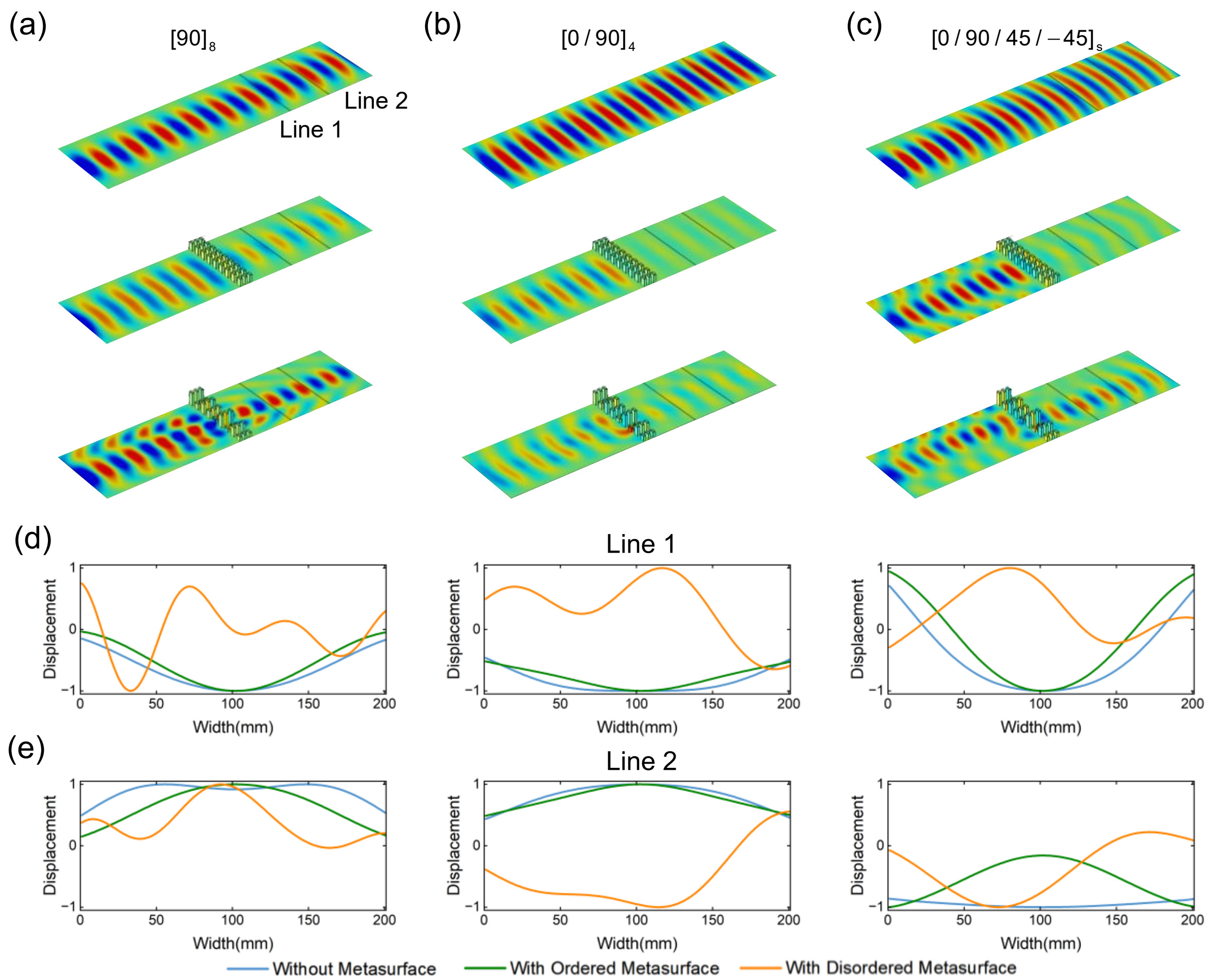

Figure S2. (a)-(c) Displacement fields for three laminate configurations, specifically unidirectional (a), cross-ply (b), and quasi-isotropic (c), without metasurfaces (top), with designed ordered metasurfaces (middle), and with disordered metasurfaces (bottom). (d)-(f) Comparison of normalized displacements along two observation lines for laminates without a metasurface (blue), with an ordered metasurface (green), and with a disordered metasurface (yellow).

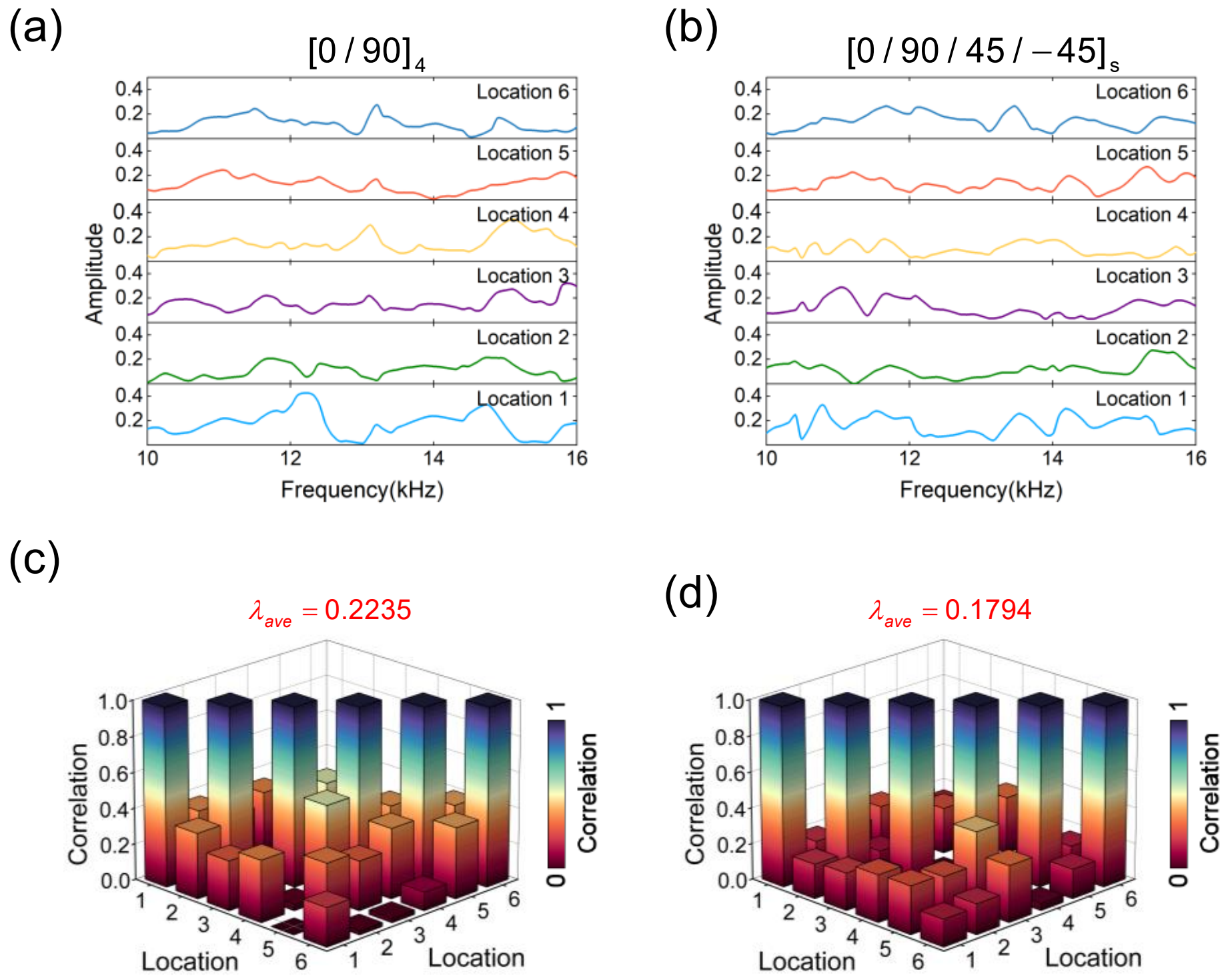

Figure S3. (a), (b) Simulated frequency response functions (FRFs) of 6 excitation sources in cross-ply and quasi-isotropic laminates, respectively. (c), (d) Correlation distributions among the 6 vibration sources.

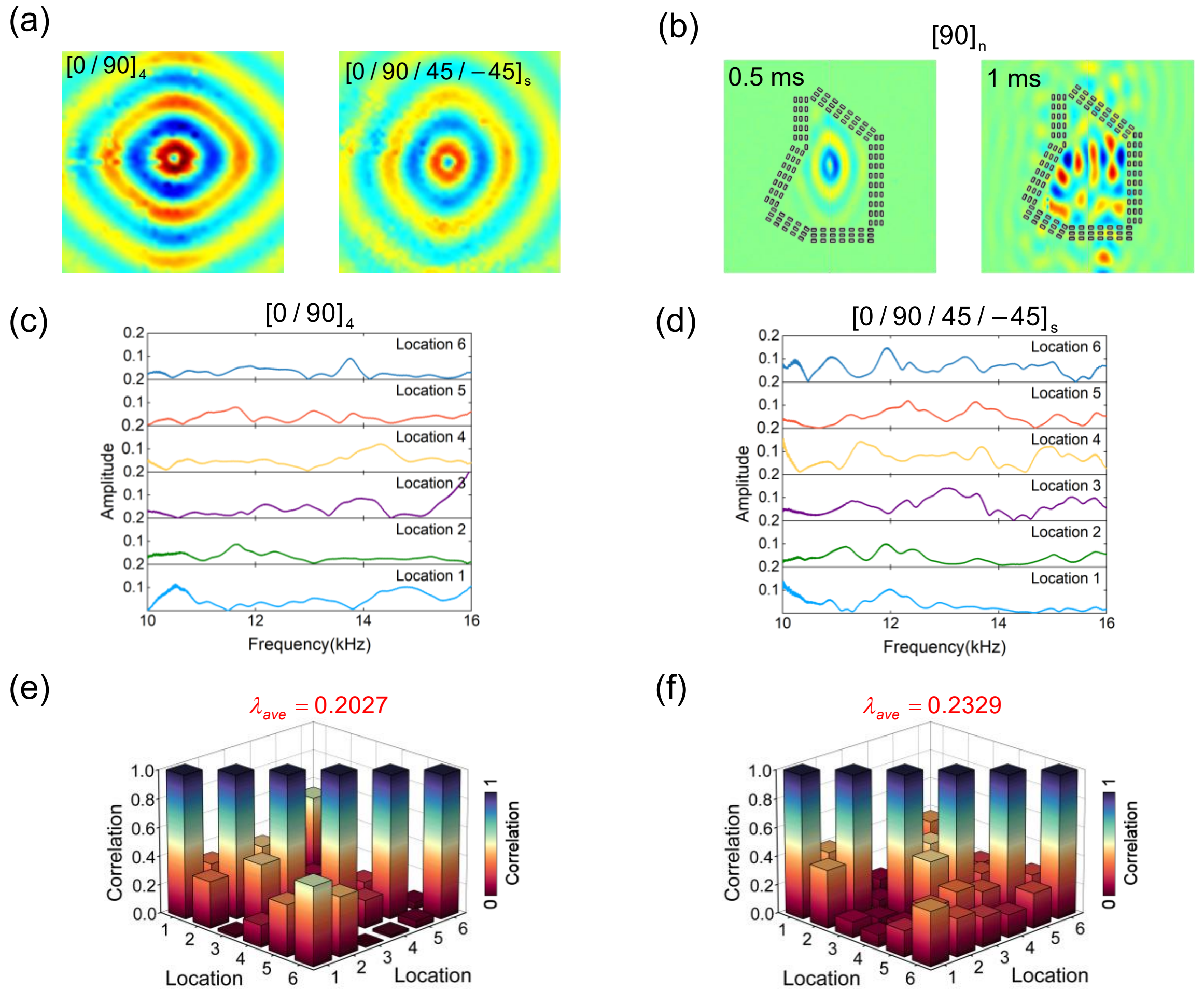

Figure S4. (a) Experimentally measured wavefield distributions in cross-ply(left) and quasi-isotropic (right) laminates. (b) Experimental wavefield distributions of the unidirectional laminate with the designed disordered metasurface at 0.5 ms and 1 ms. (c) and (d) Experimentally measured FRFs of six excitation sources for cross-ply(c) and quasi-isotropic (d) laminates, respectively. (e), (f) Correlation distributions among the excitation sources.

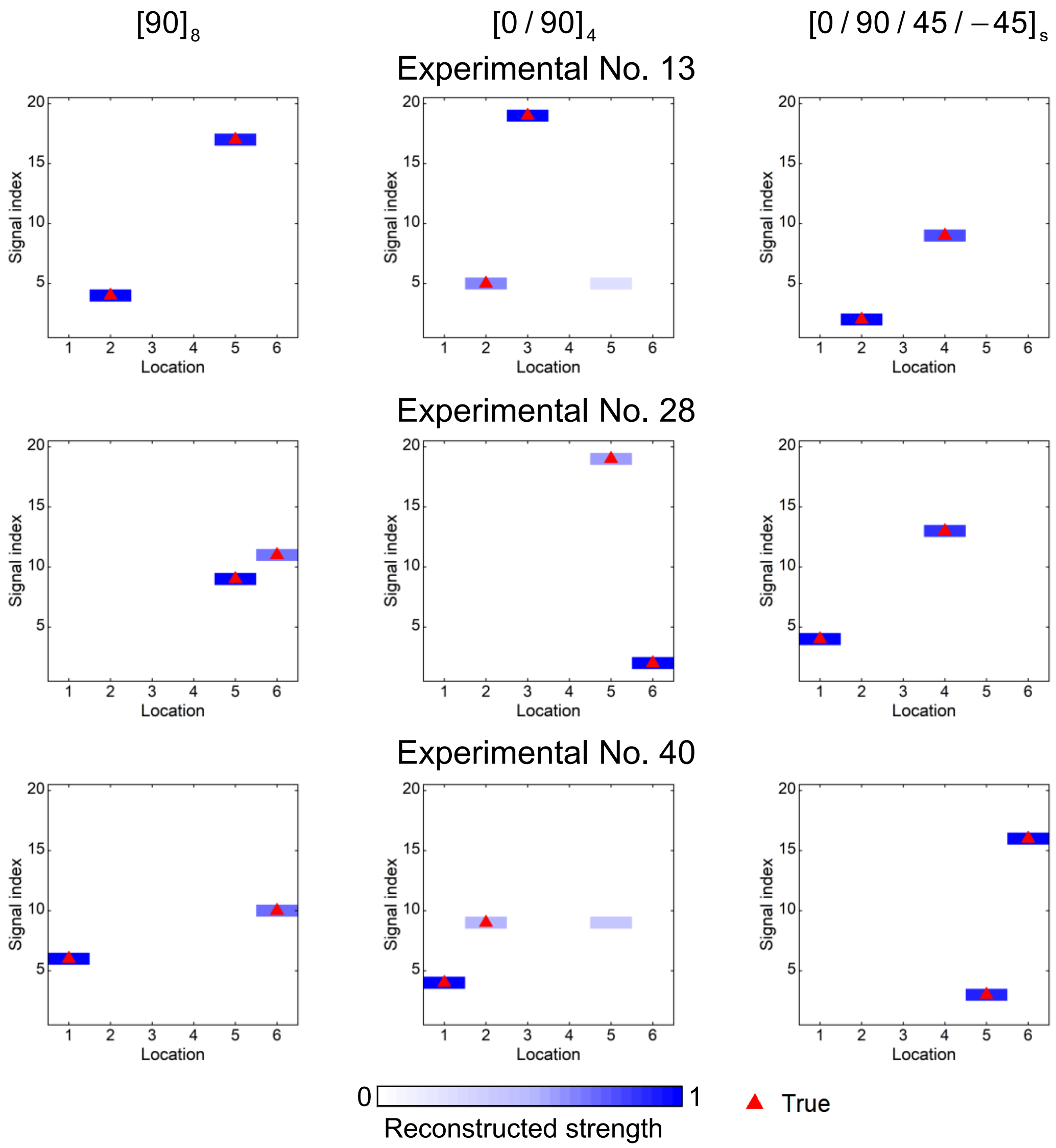

Figure S5. Reconstruction details of the 13th, 28th, and 40th experiments.

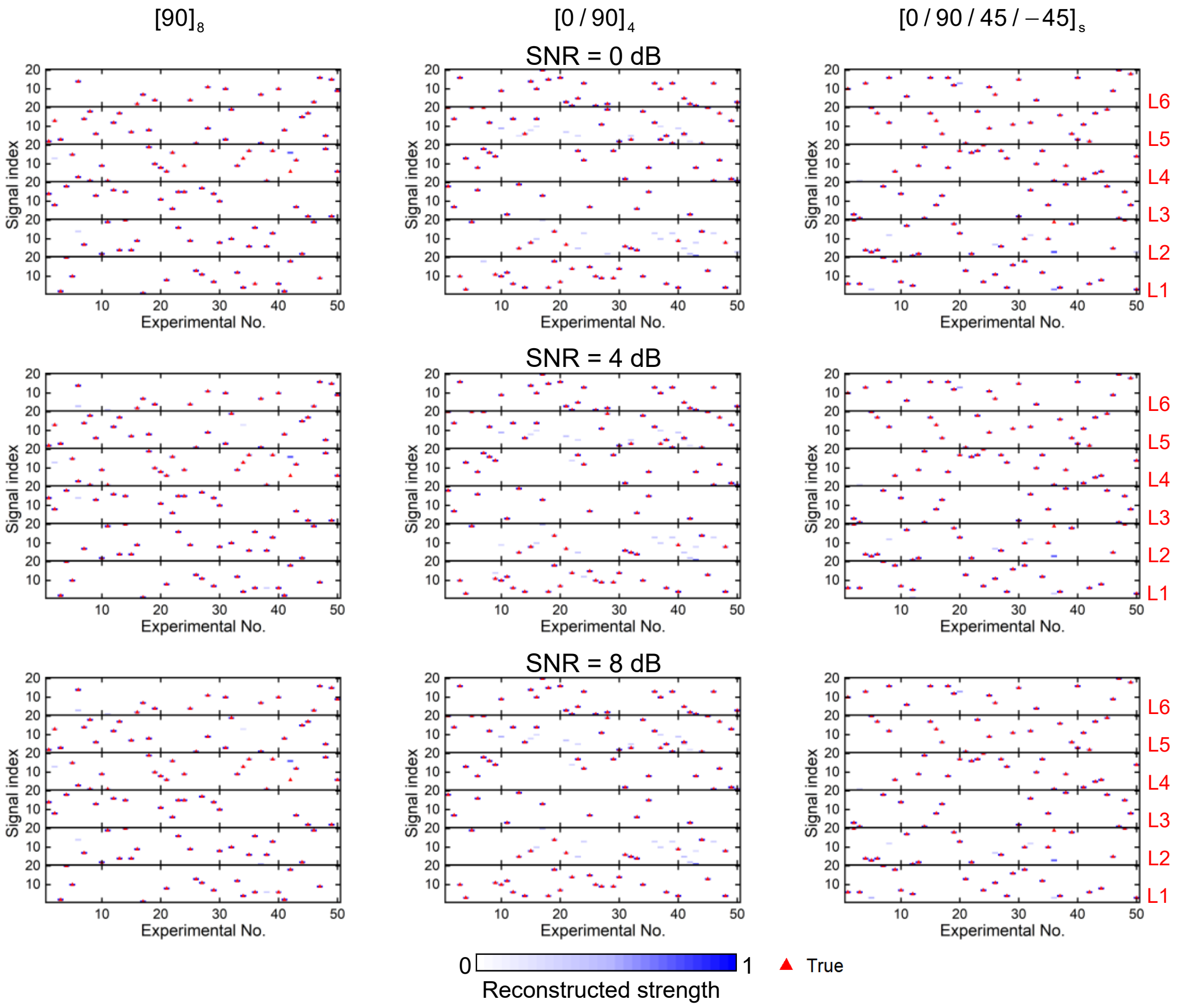

Figure S6. Two source sensing results under SNR = 0 dB, 4 dB, and 8 dB.

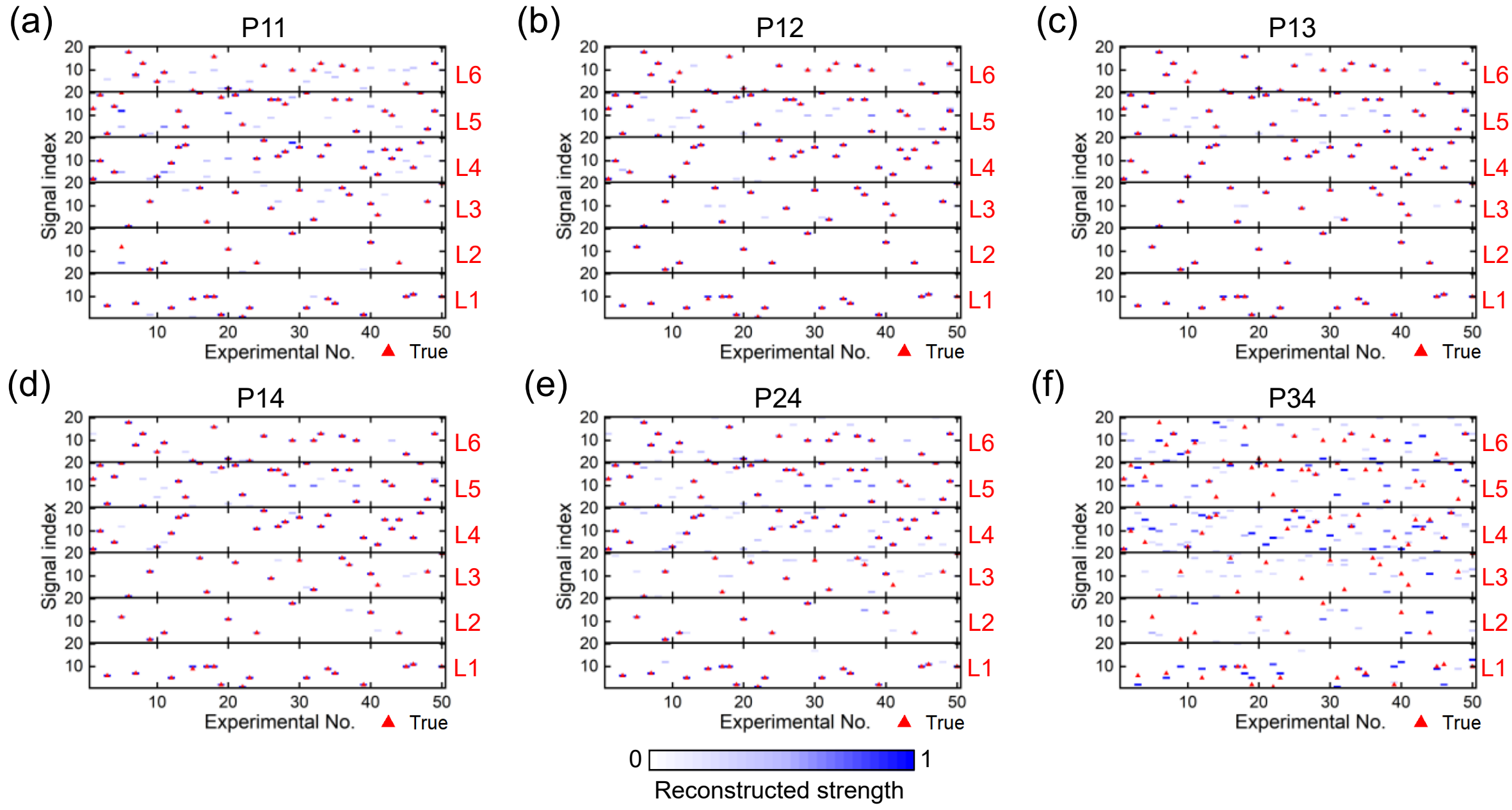

Figure S7. Two source sensing results under different sensing positions.

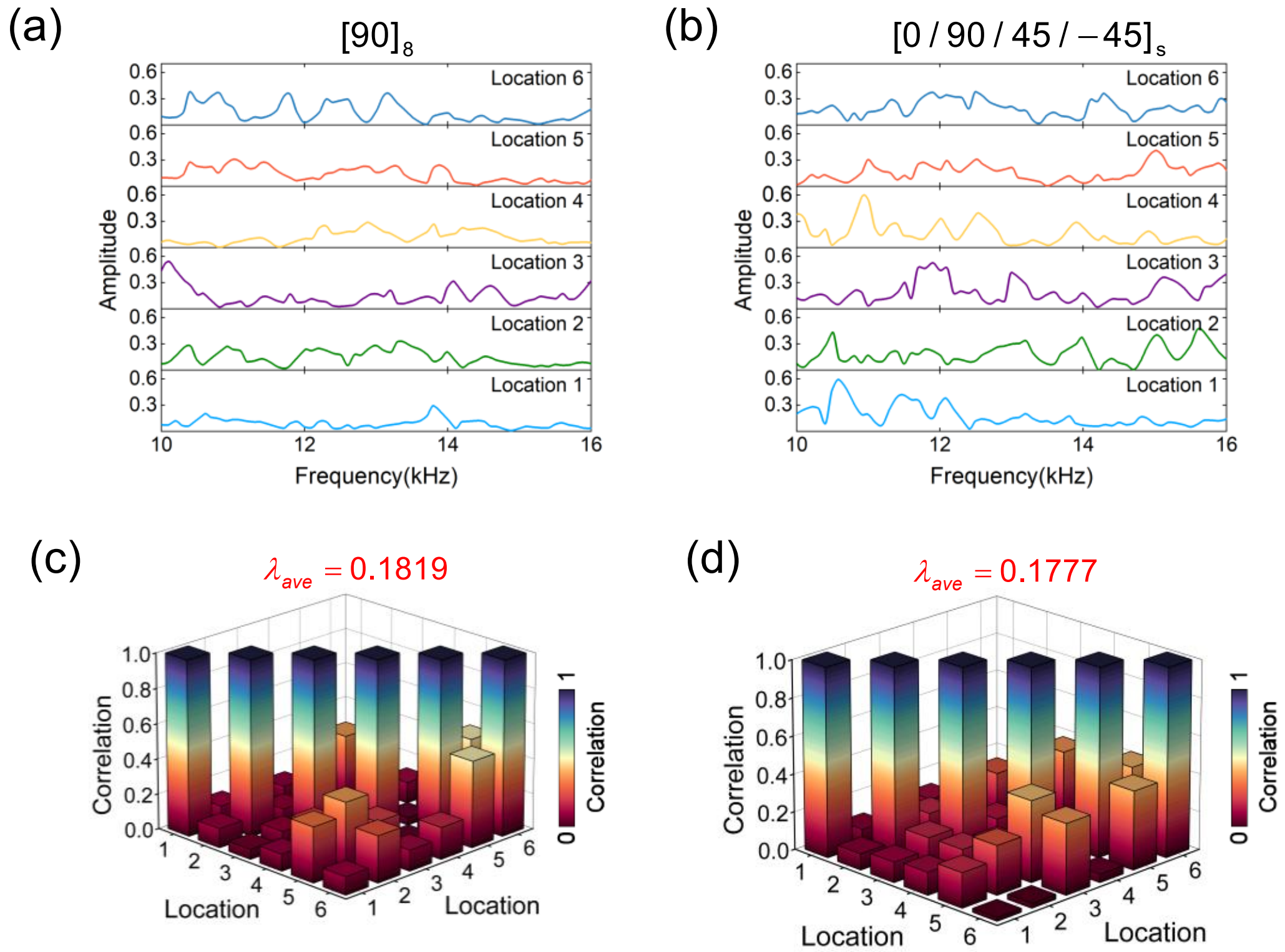

Figure S8. Simulated FRFs of the six excitation sources and the corresponding source correlation coefficient distributions for unidirectional and quasi-isotropic shell structure.

Supplementary Note 1. Equivalent material properties of the composite laminate

The composite material used in this work was T700/QY8911, and the properties of a single ply are listed in Table S1. In the laminate, the z-axis is defined as normal to the midplane formed by the x- and y-axes. For guided waves propagating in any direction within the plate, the propagation direction is defined as counterclockwise relative to the x-axis. In the global coordinate system, the stress-strain relationship for an orthotropic material with the xy plane as the plane of symmetry is expressed as follows:

$$\begin{Bmatrix}\sigma_x\\ \sigma_y\\ \sigma_z\\ \tau_{yz}\\ \tau_{xz}\\ \tau_{xy}\end{Bmatrix}=\begin{bmatrix}Q_{11} & Q_{12} & Q_{13} & 0 & 0 & 0\\ Q_{21} & Q_{22} & Q_{23} & 0 & 0 & 0\\ Q_{13} & Q_{23} & Q_{33} & 0 & 0 & 0\\ 0 & 0 & 0 & Q_{44} & 0 & 0\\ 0 & 0 & 0 & 0 & Q_{55} & 0\\ 0 & 0 & 0 & 0 & 0 & Q_{66}\end{bmatrix}\begin{Bmatrix}\varepsilon_x\\ \varepsilon_y\\ \varepsilon_z\\ \gamma_{yz}\\ \gamma_{xz}\\ \gamma_{xy}\end{Bmatrix}. \tag{S1}$$

Table S1. Material properties of a single ply of T700/QY8911

E_1(GPa)	E_2(GPa)	E_3(GPa)	G_{12}(GPa)	G_{13}(GPa)	G_{23}(GPa)	ν_{12}	ν_{13}	ν_{23}
135	9.12	9.12	5.67	5.67	5.5	0.31	0.28	0.31

When the principal material axes of individual plies in a composite laminate are not aligned with the global coordinate axes, the stiffness matrix defined in the material coordinate system can be transformed to the global coordinate system. In this work, since the z-axis of the material coordinate system coincides with that of the global system, only a rotation in the xy plane due to ply orientation is considered. Accordingly, the transformation matrix T from the material coordinate system to the global coordinate system can be simplified as shown in Equation (S2):

$$[T]=\begin{bmatrix}m^2 & n^2 & 0 & 0 & 0 & 2mn\\ n^2 & m^2 & 0 & 0 & 0 & -2mn\\ 0 & 0 & 1 & 0 & 0 & 0\\ 0 & 0 & 0 & m & -n & 0\\ 0 & 0 & 0 & n & m & 0\\ -mn & mn & 0 & 0 & 0 & m^2-n^2\end{bmatrix}, \tag{S2}$$

here, $m=\cos\theta$, $n=\sin\theta$, with θ denoting the ply orientation angle. Accordingly, the stress and strain in the global coordinate system are related to those in the material coordinate system as follows:

$$\begin{Bmatrix} \sigma_x \\ \sigma_y \\ \sigma_z \\ \tau_{yz} \\ \tau_{xz} \\ \tau_{xy} \end{Bmatrix} = [T]^{-1} \begin{Bmatrix} \sigma_1 \\ \sigma_2 \\ \sigma_3 \\ \tau_{23} \\ \tau_{13} \\ \tau_{12} \end{Bmatrix}, \tag{S3}$$

$$\begin{Bmatrix} \varepsilon_x \\ \varepsilon_y \\ \varepsilon_z \\ \frac{1}{2}\gamma_{yz} \\ \frac{1}{2}\gamma_{xz} \\ \frac{1}{2}\gamma_{xy} \end{Bmatrix} = [T]^{-1} \begin{Bmatrix} \varepsilon_1 \\ \varepsilon_2 \\ \varepsilon_3 \\ \frac{1}{2}\gamma_{23} \\ \frac{1}{2}\gamma_{13} \\ \frac{1}{2}\gamma_{12} \end{Bmatrix}. \tag{S4}$$

To transform the stiffness matrix from the material coordinate system to the global coordinate system, the Routh matrix is introduced, as shown in Equation (S5):

$$[R] = \begin{bmatrix} 1 & 0 & 0 & 0 & 0 & 0 \\ 0 & 1 & 0 & 0 & 0 & 0 \\ 0 & 0 & 1 & 0 & 0 & 0 \\ 0 & 0 & 0 & 2 & 0 & 0 \\ 0 & 0 & 0 & 0 & 2 & 0 \\ 0 & 0 & 0 & 0 & 0 & 2 \end{bmatrix}. \tag{S5}$$

By combining Equations (S3)-(S5), the stiffness matrix expressed in the global coordinate system is obtained as follows:

$$[\overline{Q}_{ij}]_k = [T]^{-1}[Q]_k[R][T][R]^{-1} = \begin{bmatrix} \overline{Q}_{11} & \overline{Q}_{12} & \overline{Q}_{13} & 0 & 0 & \overline{Q}_{16} \\ \overline{Q}_{21} & \overline{Q}_{22} & \overline{Q}_{23} & 0 & 0 & \overline{Q}_{26} \\ \overline{Q}_{31} & \overline{Q}_{32} & \overline{Q}_{33} & 0 & 0 & \overline{Q}_{36} \\ 0 & 0 & 0 & \overline{Q}_{44} & \overline{Q}_{45} & 0 \\ 0 & 0 & 0 & \overline{Q}_{54} & \overline{Q}_{55} & 0 \\ \overline{Q}_{61} & \overline{Q}_{62} & \overline{Q}_{63} & 0 & 0 & \overline{Q}_{66} \end{bmatrix}, \tag{S6}$$

here, $[Q]_k$ denotes the stiffness matrix of the k-th ply, and $[\overline{Q}_{ij}]_k$ represents the stiffness matrix of the k-th ply in the global coordinate system. For a laminate composed of plies with different orientations, the equivalent stiffness can be calculated using Equation (S7):

$$[\overline{Q}_{ij}] = \sum_{k=1}^{m} \frac{t_k}{D} [\overline{Q}_{ij}]_k, \tag{S7}$$

here, t_k is the thickness of the k-th ply, D is the total thickness of the laminate, and m denotes the total number of layers.

In this work, the laminate configurations were designed as: ① unidirectional ($[90]_8$), ② cross-ply ($[0/90]_4$), and ③ quasi-isotropic ($[0/90/-45/45]_s$). For each laminate, all layers have the same thickness, and the total laminate thickness is 1.5 mm. The equivalent material properties calculated according to Equation (S7) are listed in Table S2.

Table S2. Equivalent material properties for the three laminate configurations

Laminate	$\overline{E}_1$(GPa)	$\overline{E}_2$(GPa)	$\overline{E}_3$(GPa)	$\overline{G}_{12}$(GPa)	$\overline{G}_{13}$(GPa)	$\overline{G}_{23}$(GPa)	$\overline{v}_{12}$	$\overline{v}_{13}$	$\overline{v}_{23}$
①	9.12	135	9.12	5.67	5.5	5.67	0.02	0.28	0.31
②	72.45	72.45	9.71	5.67	5.59	5.59	0.04	0.33	0.33
③	52.73	52.73	9.71	20.27	5.59	5.59	0.3	0.24	0.24

Supplementary Note 2. Quantitative assessment of guided wave anisotropy

To quantitatively assess the capability of the designed disordered metasurface to effectively break the directional dependence of anisotropic guided waves, a displacement amplitude coefficient was defined as $\eta_i = 20log(w_i/w_{min})$, where w_i is the displacement at the measurement point under excitation from the i-th direction, and w_{min} is the minimum displacement among the six directions. The variation of η with excitation locations for the laminates with and without the metasurface is summarized in Table S3.

Table S3. Displacement amplitude coefficients η for laminates with and without the metasurface

Laminate	Metasurface	η_1	η_2	η_3	η_4	η_5	η_6
$[90]_8$	without	5.125	0.145	0.063	4.976	0.036	0
	with	0	7.049	6.973	9.880	14.019	4.882
$[0/90]_4$	without	4.526	0	8.949	7.166	4.248	1.098
	with	0	13.147	16.221	17.669	9.610	3.951
$[0/90/-45/45]_s$	without	0	0.516	0.476	0.008	0.461	0.509
	with	6.458	0	2.296	2.817	5.960	9.412

Values of η that are close indicate that the wavefield displacement in the corresponding directions is similar. For the laminates designed in this work, the displacement at symmetric locations is expected to be comparable, i.e., η values are similar for positions 1 and 4, as well

as for positions 2, 3, 5, and 6. Comparing the results with and without the metasurface, it can be observed that the introduction of the designed disordered metasurface completely disrupts the η distribution, no longer following the inherent directional dependence of the laminate. These results quantitatively demonstrate that the proposed disordered metasurface can effectively mitigate the anisotropy of guided waves in laminated plates.

Supplementary Note 3. Correlation comparison among different metasurface designs

To quantitatively evaluate the effectiveness of the proposed disordered metasurface in reducing the correlation of the sensing system, correlation analyses were performed on the multi-source responses of three different laminate configurations and compared with cases without a metasurface, as well as with periodic, quasi-periodic, and fully random metasurfaces. The corresponding correlation coefficient distributions are shown in Figure S9.

As presented in Figure S9(a), The simulation results show that without a metasurface, the average correlation coefficients of the system are all higher than 0.5, indicating strong correlations among the responses generated by different wave sources, which cannot satisfy the requirement of low correlation in the measurement matrix for compressed sensing. After introducing a regularly arranged metasurface, the periodic structure applies similar modulation effects to waves from different sources, resulting in increased correlations among the responses corresponding to different excitation sources in the measurement matrix. Furthermore, while maintaining the overall irregular boundary of the metasurface, comparative analyses were conducted for metasurface designs with periodic, quasi-periodic, and fully random height distributions (generated by random numbers), as shown in Figure S9(b)-(e). The corresponding distributions of correlation coefficients are presented in Figure S9(f)-(h), respectively. With increasing randomness in the unit-cell height distribution, the correlations among responses from different excitation sources exhibit an overall decreasing trend, indicating that the disordered design can effectively enhance the diversity of wavefield responses and reduce the correlation of the measurement matrix.

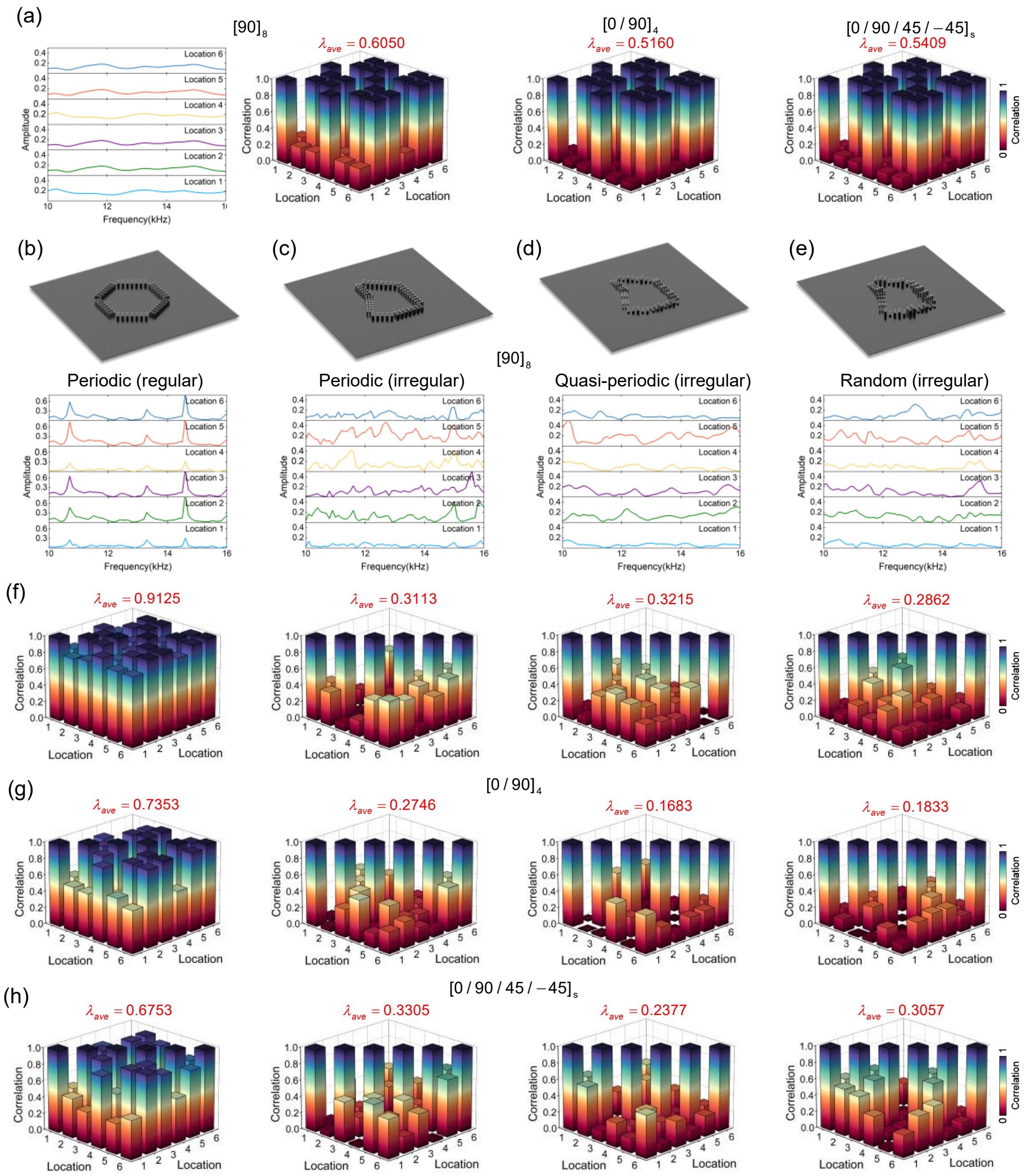

Figure S9. Comparison of correlation characteristics for different metasurface designs. (a) FRF ([90]$_8$) and correlation coefficient distribution without a metasurface. (b)-(e) Different metasurface structures and their corresponding FRFs ([90]$_8$), including regularly arranged periodic metasurfaces (b), irregularly arranged periodic metasurfaces (c), quasi-periodic metasurfaces (d), and fully random metasurfaces (e). (f)-(h) Correlation coefficient distributions of the four metasurface designs under three different laminate configurations.

However, due to the distinct anisotropic characteristics of different laminate

configurations, none of these designs can consistently achieve $\lambda_{ave} < 0.3$ across all laminate conditions. In contrast, the disordered metasurface proposed in this work is designed by considering the anisotropic wave propagation characteristics of composite materials, enabling very low correlation responses for all the three laminate configurations. This demonstrates its superior adaptability to different stacking sequences and provides an effective strategy for constructing low-correlation measurement matrices that meet the requirements of compressed sensing.

Supplementary Note 4. Details of the vibration sensing procedure

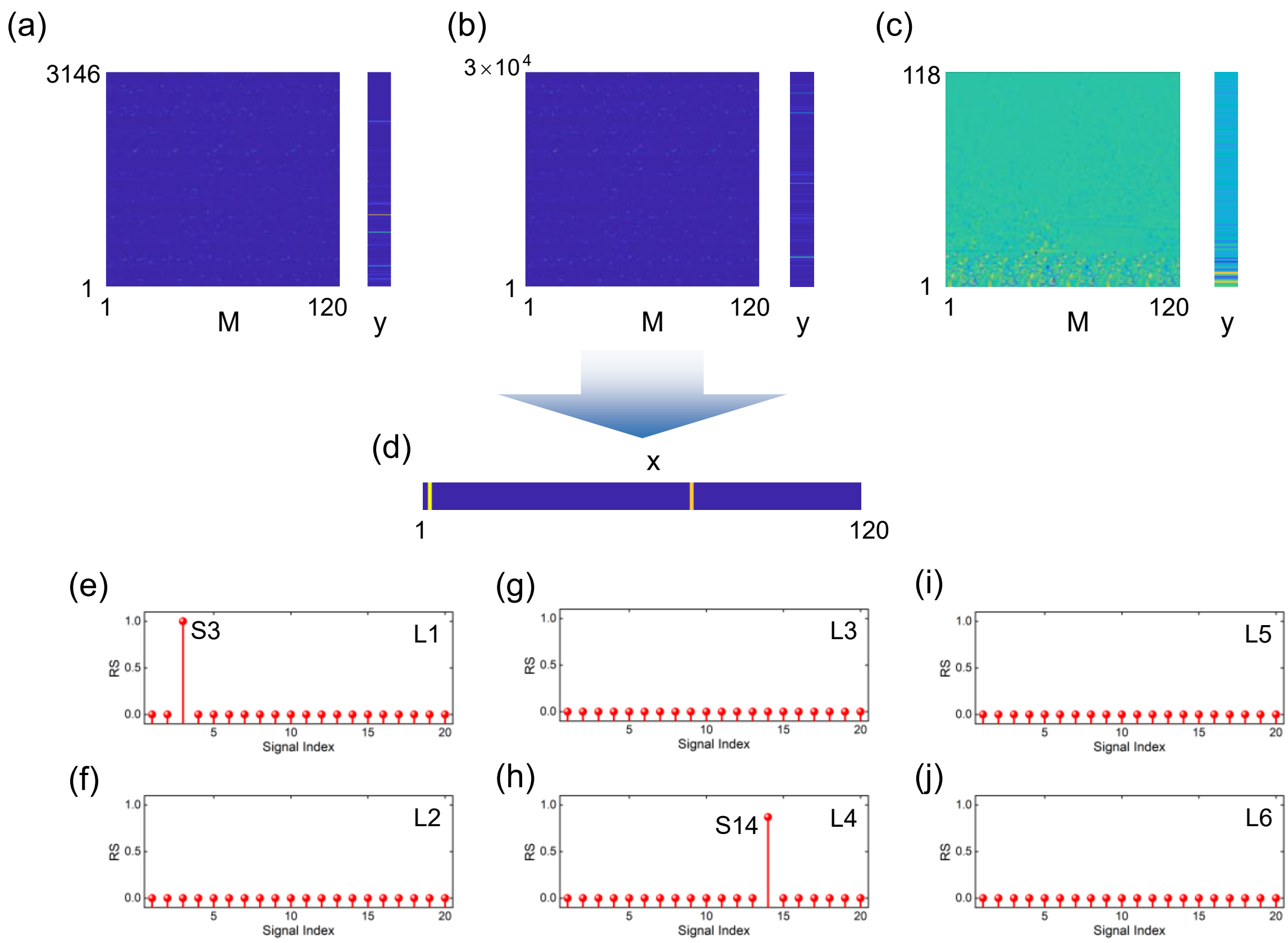

Figure S10. Schematic of the vibration sensing experimental procedure. (a) Measurement matrix **M** and measurement vector **y**. (b) Replicated **M** and **y** for information enhancement. (c) PCA compressed **M** and **y**. (d) Compressed sensing reconstruction results. (e)-(j) Detailed views of the reconstruction results in (d).

In the compressed sensing (CS) framework, the measurement matrix $M_{k\times l}$ and the measurement vector $y_{k\times 1}$ are obtained either from simulations or experimental measurements. In the simulations, $M_{k\times l}$ is obtained by multiplying the frequency response functions $W_m(\omega_k)$ of 6 excitation sources with the spectral amplitudes $S_n(\omega_k)$ of 20 random signals.

In experiments, the 20 random signals are sequentially applied to 6 locations, and the laser vibrometer collects the signals at the sensing point after modulation by the disordered metasurface. The measured signals are transformed into the frequency domain via FFT, and the spectral components within the 10-16 kHz are extracted to construct $M_{k\times l}$. The vector $y_{k\times 1}$ is obtained by simultaneously exciting arbitrary positions with different random signals, and the signals at the sensing point are collected and processed via FFT, as shown in Figure S8(a), where $k = 3146$ and $l = 120$. Subsequently, $M_{k\times l}$ and $y_{k\times 1}$ are replicated p times along the rows to enhance the representation capability of informative features in the subsequent dimensionality reduction process, increasing k to 3×10^4, as illustrated in Figure S10(b). Principal component analysis (PCA) is then performed, and the first 118 principal components are retained to reduce the dimensionality of the measurement matrix. The compressed M and y are shown in Figure S10(c). *Finally, the reduced sensing matrix and measurement vector are used to construct the inverse problem of compressive sensing. For the compressive sensing formulation* $y = Mx$, *the target vector* x *is reconstructed via L1-norm minimization, as expressed in Eq. (S8):*

$$\tilde{x} = min\|x\|_1, s.t\ y = Mx. \qquad \text{(S8)}$$

In the above equation, $\tilde{x}$ denotes the optimal estimate of the target vector x, and $min\|x\|_1$ represents the L1-norm of x. The inverse problem is solved using the two-step iterative shrinkage/thresholding (TwIST) algorithm, which is employed to obtain a sparse solution by minimizing the discrepancy between the measured responses and the reconstructed signals. The reconstructed vector x is shown in Fig. S10(d), where the magnitude of each element represents the reconstruction strength associated with each vibration source location and excitation signal. This enables the identification of the number of sources, their locations, and the corresponding excitation signals. For visualization, x is reshaped into a 6×20 matrix, and the corresponding localization results are presented in Fig. S10(e)-(j). The results indicate that the vibration sources are located at positions 1 and 4, excited by signals 3 and 14, respectively, which is consistent with the ground truth, confirming the correctness of the reconstruction results.

Supplementary Note 5. Comparison of sensing accuracy among different metasurface designs

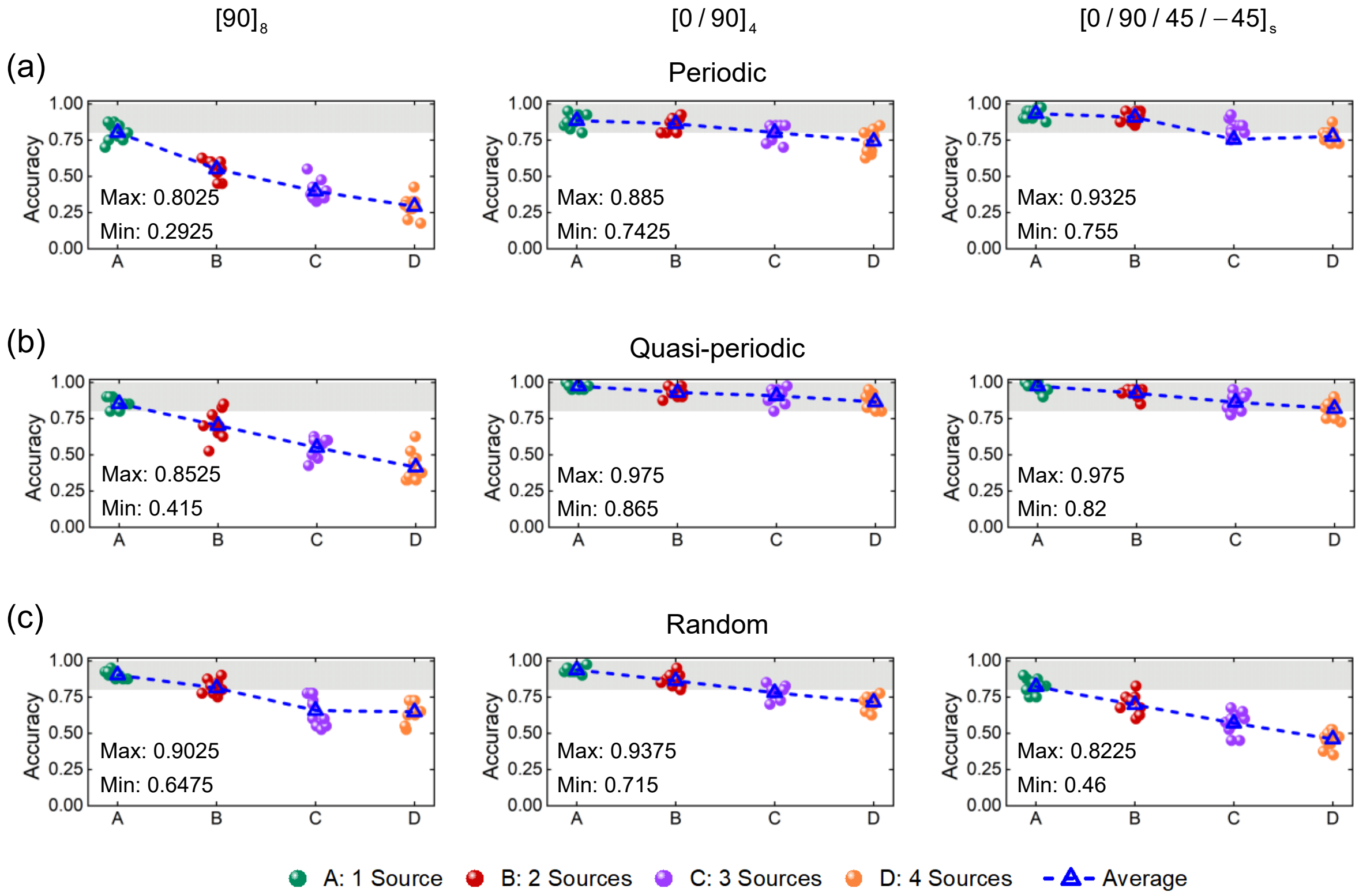

Figure S11. Comparison of vibration sensing accuracy for different metasurface designs. (a) Periodic metasurface. (b) Quasi-periodic metasurface. (c) Random metasurface.

In ***Supplementary Note 3***, we quantitatively analyze the correlation characteristics of different metasurface designs. To further intuitively evaluate the influence of different designs on sensing performance, periodic, quasi-periodic, and random metasurface designs (with unit-cell heights generated by random numbers) were constructed while maintaining the overall irregular boundary of the metasurface, and the corresponding vibration sensing tests were conducted (Figure S11).

For the unidirectional laminate, the single source localization accuracy under all three metasurface designs remained above 80%. However, as the number of excitation sources increases, the sensing accuracy decreases to different extents. Notably, although the randomly designed metasurface exhibits a certain degree of decorrelation capability in the correlation analysis, its localization performance is still unsatisfactory. This is mainly attributed to the strong anisotropy of the unidirectional laminate, which leads to a significant difference in the

response amplitude distributions generated by excitations at different locations. As shown in Figure S9(e), the response amplitude corresponding to location 1 is significantly lower than those of other locations. Such weak responses are more likely to be masked by signals from other sources during signal reconstruction, thereby reducing localization accuracy. Therefore, reduced correlation does not necessarily correspond directly to improved localization accuracy, and variations in response amplitude distributions also play an important role in the effective reconstruction of multi-source signals.

Similarly, under periodic and quasi-periodic metasurface designs, the sensing performance of the unidirectional laminate remains affected by the non-uniform response amplitude distribution induced by its strong anisotropy. In contrast, the anisotropy of orthogonal and quasi-isotropic laminates is relatively weaker, resulting in more balanced response amplitude distributions among different excitation locations. Consequently, the sensing accuracy exhibits a better correspondence with the trend of correlation reduction. Specifically, when $\lambda_{ave} < 0.3$, both single source and two sources sensing results maintain accuracies above 80%. Compared with the aforementioned designs, the disordered metasurface proposed in this work not only reduces the correlation among responses but also avoids the response homogenization caused by regular structures, thereby enabling more stable multi-source signal reconstruction in laminate structures with different degrees of anisotropy.

Supplementary Note 6. Principal component analysis of experimental data

In the sensing experiments, the signals collected were first transformed via FFT to construct the observation matrix $M_{k\times l}$. However, this matrix contains redundant information and noise. To enhance signal identification, Principal Component Analysis (PCA) was applied for dimensionality reduction. After acquiring the measurement vector $y_{k\times 1}$, both $M_{k\times l}$ and $y_{k\times 1}$ were replicated p times along the rows to enhance the representation capability of effective features during the subsequent dimensionality reduction process. The two matrices were then mean centered, and PCA was performed on $M_{kp\times l}$ to compute the principal component loadings $\Upsilon_{l\times kp}$. The first q components were retained to construct $\Upsilon_{l\times q}$, resulting in the compressed observation matrix $M'_{l\times q} = M^T_{kp\times l} \cdot \Upsilon_{l\times q}$ and measurement vector $y'_{1\times q} =$

$y^T_{kp\times 1} \cdot Y_{l\times q}$.

To evaluate the influence of the replication factor (p) on sensing performance, a parameter sensitivity analysis was further conducted, with the results summarized in Table S4. The results indicate that the sensing accuracy shows a slight improvement as the number of replications increases from 1 to 10. However, when the replication factor is further increased beyond 10, the sensing performance exhibits a mild decline. Therefore, a replication factor of $p = 10$ is ultimately selected in this work to achieve an improved sensing performance.

Table S4. Effect of replication factor p on sensing performance

Replication factor	Sensing accuracy		
	$[90]_8$	$[0/90]_4$	$[0/90/-45/45]_s$
$p = 1$	94%	88%	92%
$p = 2$	94%	88%	92%
$p = 4$	94%	88%	92%
$p = 6$	90%	88%	96%
$p = 8$	94%	88%	92%
$p = 10$	96%	96%	94%
$p = 12$	90%	88%	96%

By projecting high dimensional correlated data onto a small number of orthogonal principal components, PCA effectively reduces dimensionality, eliminates correlation, and suppresses noise, thereby improving the stability and efficiency of subsequent vibration sensing. Notably, the choice of 118 components was selected as a representative value within a stable performance regime. Comparisons with $q = 108$, $q = 114$, or without PCA show that sensing accuracy remains robust, with snesing accuracy exceeding 90% even without PCA and a further improvement after PCA processing (Table S5). These results indicate that, despite the substantial reduction in dimensionality, reconstruction performance remains high within a reasonable range of principal components, confirming the effectiveness and robustness of the PCA preprocessing.

Table S5. Sensing performance for various PCA component selections

PCA Configuration	Sensing accuracy		
	$[90]_8$	$[0/90]_4$	$[0/90/-45/45]_s$
Without PCA	90%	94%	92%
PCA, q= 108	90%	90%	92%
PCA, q= 114	94%	94%	92%
PCA, q= 118	96%	96%	94%

Supplementary Note 7. Sensitivity analysis of the reconstruction strength threshold

In this work, a reconstruction strength exceeding 0.5 is selected as the criteria for correct identification. To verify the rationality of this threshold, a threshold sensitivity analysis was further conducted. Specifically, the reconstruction strength threshold was gradually varied from 0.3 to 0.9, and the corresponding vibration sensing results were evaluated to assess the influence of threshold selection on identification accuracy. The results are presented in Table S6.

Table S6. Sensing accuracy under different reconstruction strength thresholds

Thresholds	Sensing accuracy		
	$[90]_8$	$[0/90]_4$	$[0/90/-45/45]_s$
0.3	88%	64%	88%
0.4	90%	80%	94%
0.5	96%	96%	94%
0.6	90%	90%	92%
0.7	82%	68%	76%
0.8	58%	54%	56%

The results indicate that the choice of reconstruction strength threshold has a significant influence on the vibration sensing accuracy. When a relatively low threshold (< 0.5) is adopted, the system exhibits a reduced accuracy. This is because a lower threshold retains more

reconstructed responses, including weak responses originating from non-target regions, which may be incorrectly identified as valid vibration sources and thus increase false detection rate. As the threshold increases to the range of $0.5-0.6$, the sensing accuracy improves significantly and remains consistently above 90%. In this range, the reconstruction peaks associated with true vibration sources are effectively preserved, while non-target responses caused by noise and reconstruction errors are substantially suppressed, resulting in a more reliable identification performance. When the threshold is further increased (≥ 0.7), the sensing accuracy rapidly decreases. Although a higher threshold further suppresses non-target responses, it may also eliminate target signals with relatively weak reconstruction strengths, thereby increasing the risk of missed detections. Considering both sensing accuracy and identification stability across different threshold values, a threshold of 0.5 is ultimately adopted for vibration source identification in this work.

Supplementary Note 8. Effect of source position variation on sensing performance

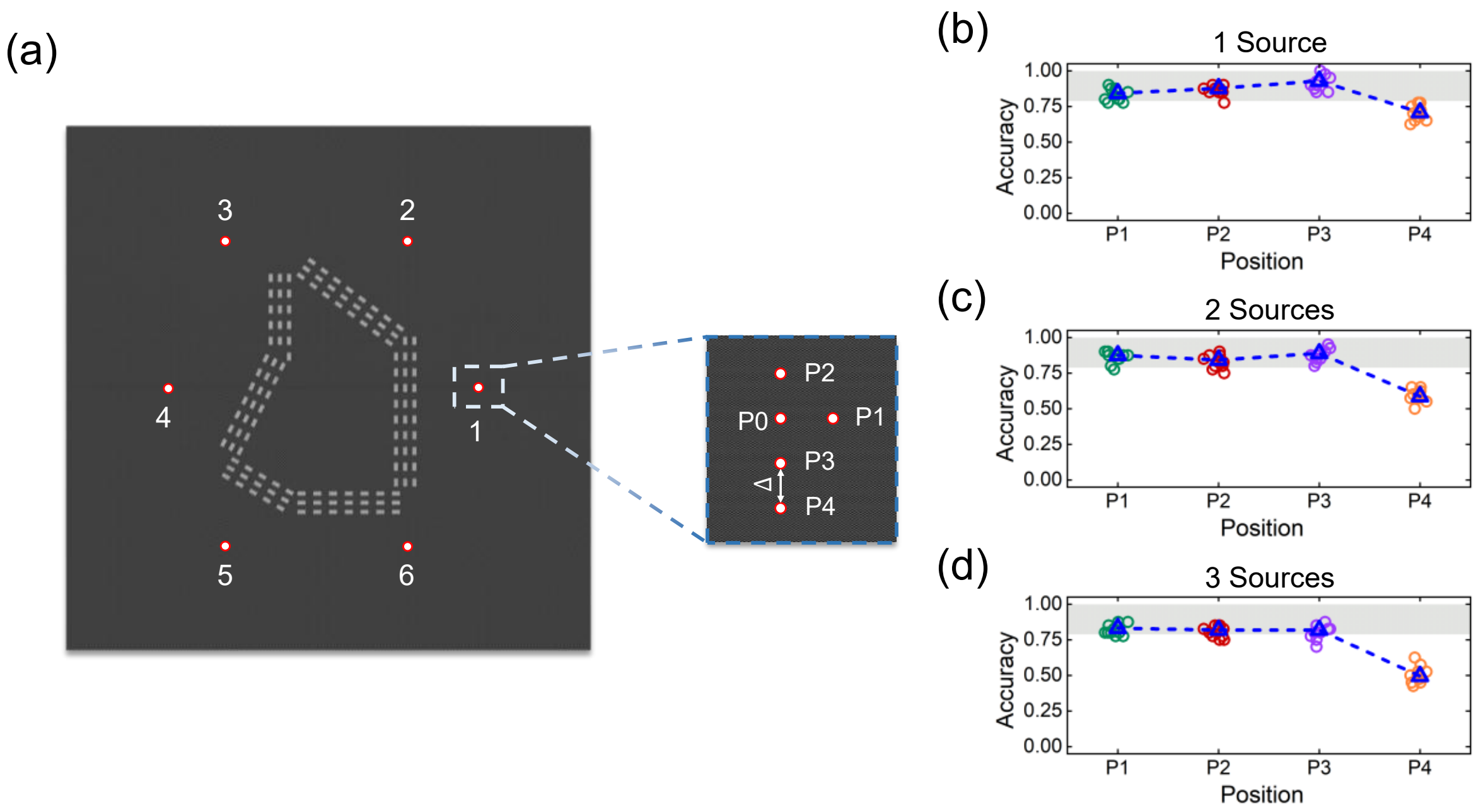

Figure S12. Sensing performance under different excitation location deviations. (a) Schematic illustration of excitation positions with different offsets from the calibrated position. (b)-(d) Sensing accuracy under single-, double-, and triple-source excitation conditions.

In practical vibration sensing scenarios, the excitation position is usually difficult to be

completely fixed at the predefined location. Therefore, it is necessary to evaluate the influence of excitation position deviation on the performance of single-sensor vibration sensing. To this end, additional sensing tests under different excitation position deviations were conducted to investigate the adaptability of the proposed SSVS system to variations in source locations.

In the numerical simulations, the frequency response functions (FRFs) of the system were first calculated based on the calibrated excitation positions, and the corresponding measurement matrix M was constructed. Subsequently, as shown in Figure S12(a), 6 excitation positions were simultaneously shifted along different directions around the calibrated excitation location, with an adjacent offset distance of $\Delta = 2$ mm. These shifted positions were used to simulate the spatial deviations between the actual excitation points and the calibrated locations during practical measurements, and the corresponding vibration responses were calculated. The 1 source, 2 sources, and 3 sources sensing results under different excitation position deviations are presented in Figure S12(b)-(d). When the excitation positions have the same offset distance (P1, P2, and P3), the sensing accuracy corresponding to different deviation directions remain generally consistent and all exceed 80%, indicating that the SSVS possesses certain adaptability to small range position variations. As the offset distance further increases, the mismatch between the measurement matrix and the actual responses becomes more significant, leading to a gradual degradation in sensing performance. When the offset distance reaches 4 mm, the single source sensing accuracy decreases to 70%.

The above results indicate that excitation position deviations introduce discrepancies between the actual responses and the calibrated measurement matrix, thereby affecting sensing performance. However, within the limited deviation range investigated, the proposed SSVS system can still maintain relatively stable vibration sensing capability, demonstrating a certain tolerance to excitation position variations.

Supplementary Note 9. Evaluation of sensing performance under different temperature

To evaluate the adaptability of the proposed SSVS system under temperature variations, the influence of temperature-induced material property changes on vibration sensing performance was further investigated over a temperature range of -20 °C to 60 °C. Since the propagation characteristics of elastic guided waves are closely related to the mechanical

properties of the medium, temperature-induced variations in material properties can directly affect guided wave velocity, dispersion characteristics, and structural frequency response features, thereby introducing discrepancies between the actual responses and the measurement matrix established under the calibrated condition.

In the numerical simulations, the effects of temperature on the key mechanical parameters of 304 stainless steel and composite materials were considered. As shown in Figure S13(a)-(b), with increasing temperature, the elastic modulus and density exhibit an overall decreasing trend, while Poisson's ratio shows a slight increase. These variations in material parameters lead to changes in the dynamic responses of the structure. To further analyze the influence of temperature variations on vibration sensing performance, the structural frequency response functions (FRFs) were recalculated under different temperature conditions, and sensing tests were conducted based on the temperature-dependent responses. It should be noted that the measurement matrix M was consistently constructed under the reference temperature condition (20 °C), simulating a practical structural monitoring scenario where environmental temperature changes occur without real-time updating of the sensing model.

The sensing results are presented in Figure S13(c)-(e). As the temperature gradually deviates from the reference temperature, the sensing accuracy decreases due to the mismatch between the actual responses and the measurement matrix constructed at 20 °C, which is induced by temperature dependent variations in material properties. However, within a moderate temperature variation range of 0 °C to 40 °C, the proposed SSVS system maintains a sensing accuracy above 80%, demonstrating its capability to tolerate temperature induced disturbances within this range. At more extreme temperatures (-20 °C and 60 °C), the larger deviations in material properties result in more significant discrepancies between the actual responses and the calibrated measurement matrix, leading to a further degradation in sensing performance.

The above results demonstrate that temperature variations affect guided wave propagation behavior by modifying the mechanical properties. Nevertheless, within a limited temperature range, the proposed SSVS system can maintain effective vibration sensing capability.

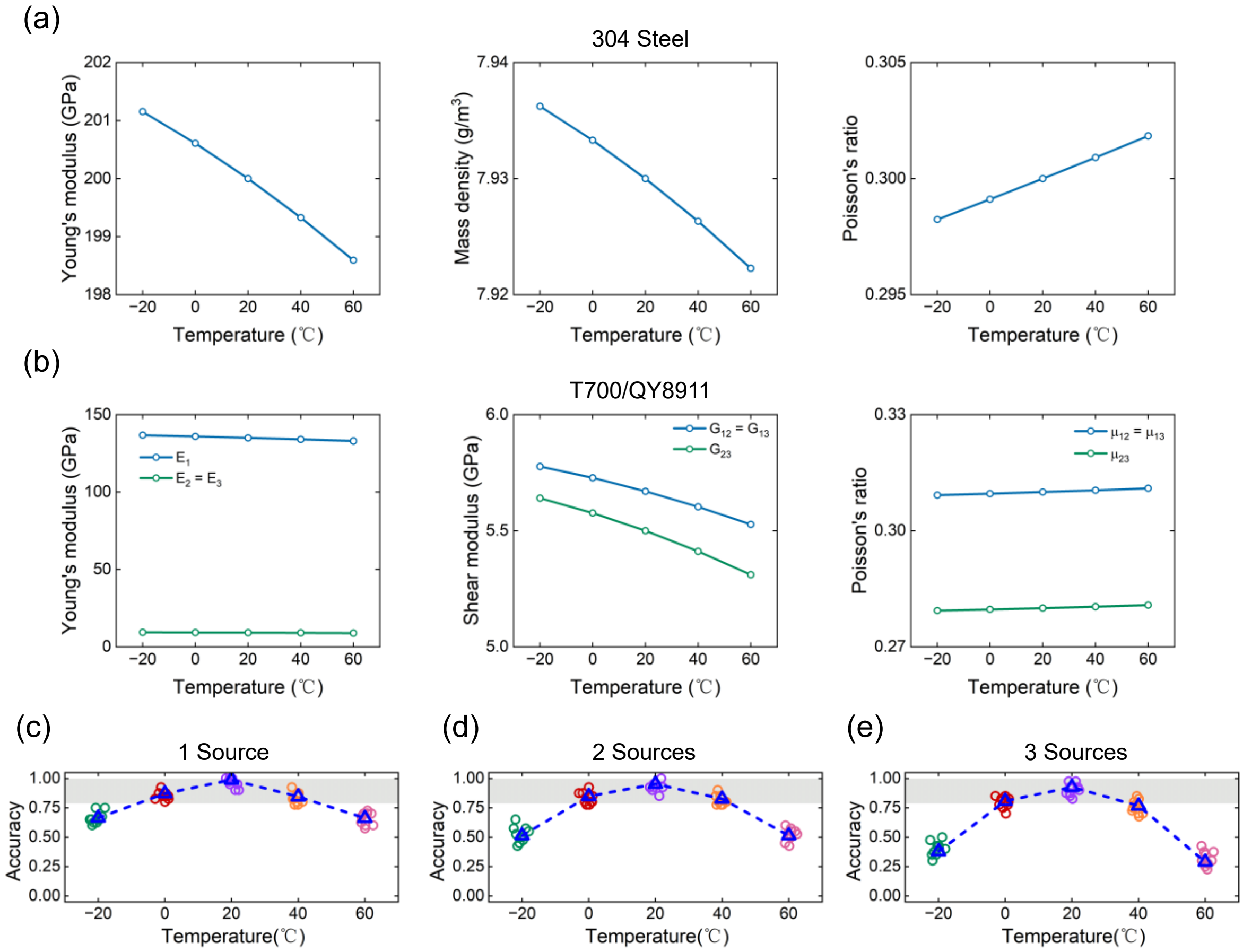

Figure S13. Evaluation of SSVS sensing performance under different operating temperatures. (a) Temperature dependent variations of the material properties of 304 stainless steel. (b) Temperature dependent variations of the material properties of the T700/QY8911 composite material. (c)-(d) Sensing accuracy of 1 source, 2 sources, and 3 sources cases under different temperature conditions.

Supplementary Note 10. Influence of metasurface bonding conditions on sensing performance

During practical service, the bonding performance between the metasurface and the composite laminate may degrade due to environmental effects. Since the adhesive layer is responsible for load transfer between the metasurface and the laminate, variations in its stiffness may further alter the dynamic response of the structure and affect the vibration sensing performance. Therefore, the sensing performance of the SSVS system under different adhesive layer stiffness conditions was further numerically analyzed. Different bonding conditions were simulated by varying the Young's modulus of the adhesive layer (E_{bond}), between the metasurface and the laminate, as shown in Figure S14(a). The thickness of the adhesive layer

was set to 0.01 mm. Taking the fully bonded condition as the reference state, the Young's modulus of the adhesive layer was varied within $10^{12} - 10^{10}$ Pa, and the phase responses of the unit cells and the structural frequency response functions (FRFs) under different adhesive layer stiffness conditions were calculated. Vibration sensing tests were then conducted based on the corresponding responses.

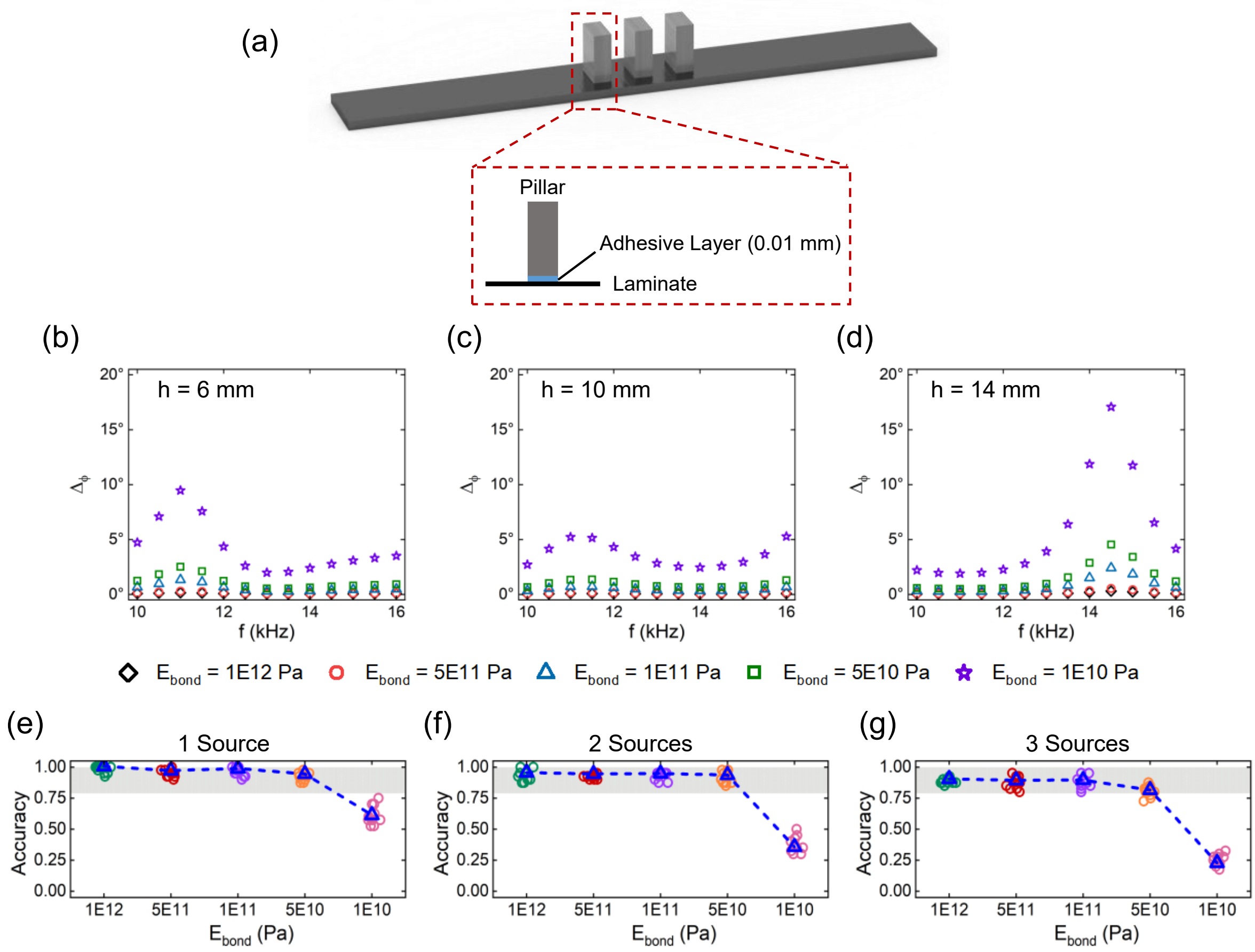

Figure S14. Effect of metasurface bonding conditions on vibration sensing performance. (a) Schematic illustration of the metasurface bonding structure. (b)-(d) Phase response shifts of metasurface unit cells with pillar heights of 6 mm, 10 mm, and 14 mm under different adhesive layer stiffness conditions. (e)-(g) Vibration sensing accuracy of the SSVS system under different adhesive stiffness conditions.

As shown in Figure S14(b)-(d), the relationships between adhesive layer stiffness variation and phase shift are presented for unit cell heights of 6 mm, 10 mm, and 14 mm, respectively. The results indicate that when the Young's modulus of the adhesive layer decreases to 5×10^{10} Pa, the phase deviation of the unit cell relative to the reference condition remains within 5°. However, when the modulus is further reduced to 10^{10} Pa, the

maximum phase deviation reaches 17°, indicating that variations in adhesive layer stiffness can influence the wave manipulation capability of the metasurface to some extent. Furthermore, vibration sensing tests were performed based on the FRFs obtained under different adhesive layer stiffness conditions, and the results are presented in Figure S14(e)-(g). When $E_{bond} \geq 5 \times 10^{10}$ Pa, the sensing accuracy of the system can still be maintained above 80%. As the adhesive layer stiffness further decreases, the mismatch between the measurement matrix and the actual responses becomes more pronounced, resulting in a significant reduction in sensing accuracy.

The above results demonstrate that variations in the stiffness of the adhesive layer between the metasurface and the laminate can influence the system response to some extent. However, within the investigated range, the proposed disordered SSVS system can still maintain effective sensing performance, providing useful insights for structural applications where adhesive properties may vary during practical service.

Supplementary Note 11. Experiments evaluation of sensing performance under different boundary conditions

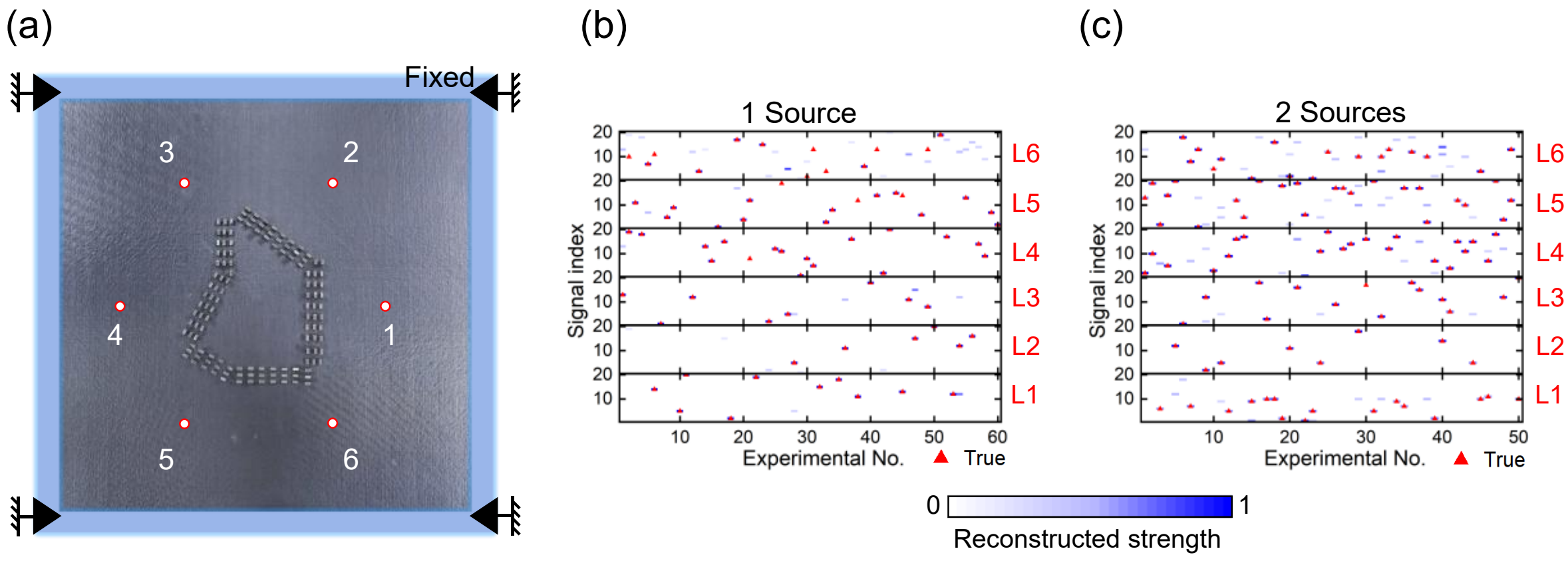

Figure S15. Effect of boundary condition variations on SSVS performance. (a) Schematic illustration of the experimental setup under four corners clamped boundary conditions. (b)-(c) single source and double sources vibration sensing results under four-corner clamped boundary conditions.

To evaluate the influence of boundary condition on the sensing performance of the SSVS system, vibration sensing experiments were conducted under four corners clamped boundary conditions. As shown in Figure S15(a), the laminate was changed from the original free

boundary condition to four corners clamped constraint, while all other experimental conditions were kept unchanged. It should be noted that the measurement matrix M constructed under the free boundary condition was still used during the sensing process, simulating a practical monitoring scenario where structural boundary conditions change without updating the sensing model.

Single source and double sources vibration sensing tests were performed under the four corners clamped condition, and the results are shown in Figure S15(b)-(c). The experimental results demonstrate that the single source sensing accuracy reaches 93.33%, while the double sources sensing accuracy is 74%. Compared with the original boundary condition, the double sources sensing performance exhibits a certain degradation. The above results indicate that variations in boundary conditions can affect sensing performance. However, under the new constraint condition, the proposed SSVS system can still achieve effective single source vibration sensing, demonstrating a certain adaptability to changes in practical boundary conditions.

Supplementary Note 12. Vibration sensing performance of the SSVS system under different curvature conditions

In composite shell structures, curvature variations may alter the geometric configuration and vibration response characteristics, thereby potentially affecting vibration sensing performance. Therefore, the sensing capability of the SSVS system under different curvature conditions was further analyzed. The shell models of a cross-ply laminate with different curvatures were numerically established. Three curvature conditions were configured by varying the central angle θ to 60°, 75°, and 90°, respectively, as shown in Figure S16(a)-(c). Under different curvature conditions, the metasurface design was kept unchanged, and the correlations of the corresponding measurement matrices were calculated. The results show that the correlation coefficients under all three curvature conditions remain below 0.3, and the corresponding correlation coefficient distributions are presented in Figure S16(d)-(f), indicating that the system can maintain low response correlations under different curvature conditions.

Subsequently, vibration sensing tests were conducted under single source and multi-

source excitation conditions. As shown in Figure S16(g)-(i), the system maintains sensing accuracies above 90% for both single source and double sources excitations under different curvature conditions. As the number of excitation points increases to three and four, the influence of curvature variations on sensing performance gradually becomes pronounced due to the increased difficulty in separating multi-source signals. Particularly under larger curvature conditions, the sensing accuracy exhibits a certain degree of degradation; however, the overall accuracy remains above 70%.

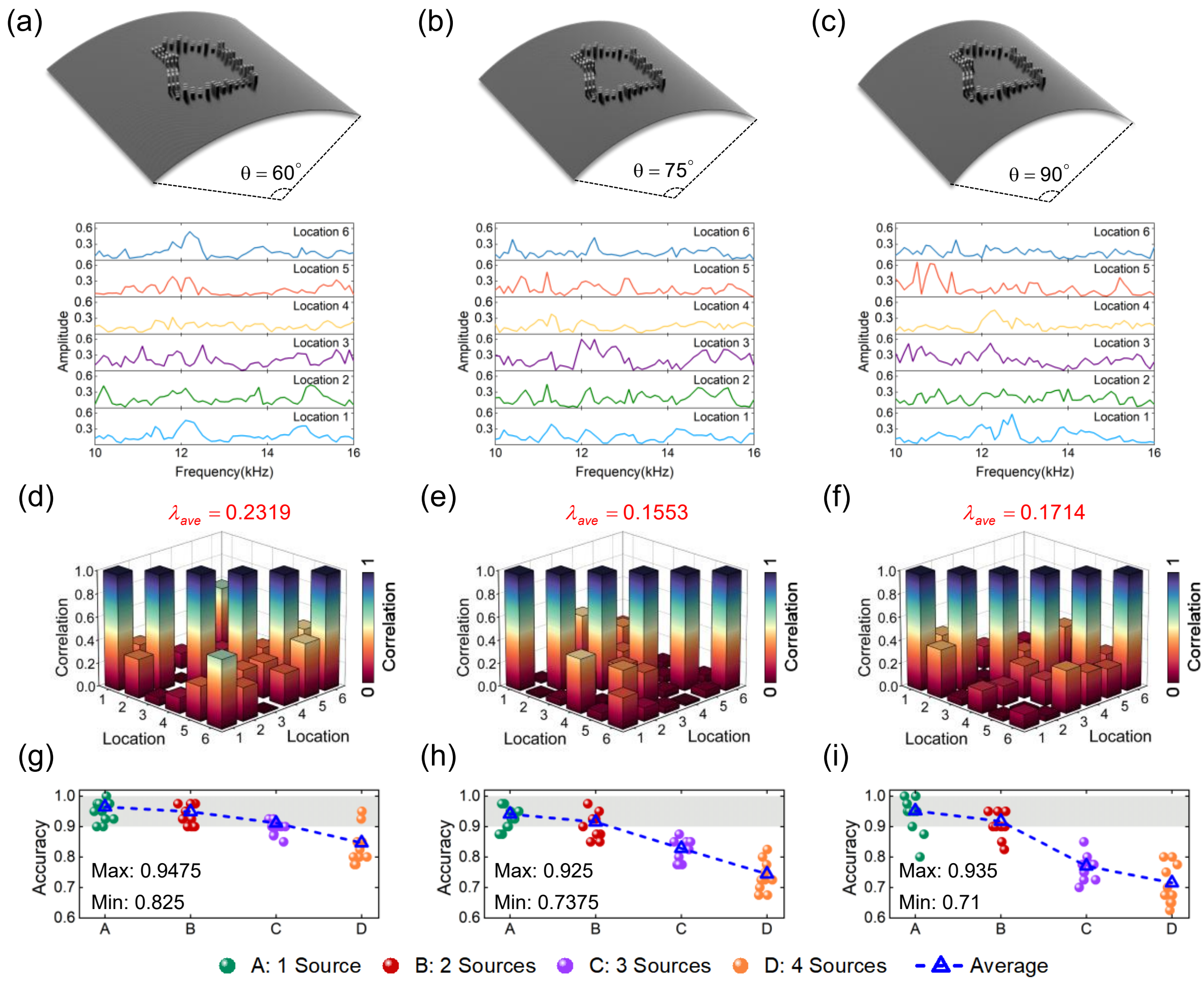

Figure S16. Vibration sensing performance of the SSVS system under different curvature conditions. (a)-(c) Schematic illustrations and FRFs of composite shell structures with different curvatures. (d)-(f) Correlation coefficient distributions under different curvature conditions. (g)-(i) Sensing accuracy under different sources conditions with different curvatures.

The above results indicate that curvature variations can influence vibration responses and increase the difficulty in multi-source vibration signal reconstruction. Nevertheless, within the

investigated curvature range and with a limited number of excitation sources, the proposed SSVS system can still achieve effective vibration sensing, demonstrating the potential applicability of this method for curved composite structures.

Supplementary Note 13. Effect of metasurface integration on structural dynamic characteristics

Since the steel pillar metasurface introduces additional mass, its integration may alter the overall dynamic characteristics of the composite structure. Therefore, the mass loading effect of the metasurface and its influence on the structural modal responses were quantitatively analyzed. First, the mass of the steel pillar metasurface used in the experiments was measured, and the corresponding mass loading was calculated. The measurement results show that the total mass of the metasurface is 153.5 g, corresponding to a mass loading of 0.614 kg/m^2. This result indicates that the designed metasurface introduces only a limited additional mass while achieving wavefield manipulation functionality.

Furthermore, to evaluate the influence of the metasurface on the dynamic characteristics of the composite structure, modal experiments were conducted on composite laminates with and without the metasurface. The experimental setup is shown in Figure S17(a). Both structures were tested under identical boundary conditions and measurement procedures to obtain their frequency response characteristics. By measuring the frequency responses at 9 blue test points and averaging the results, the structural frequency responses in the range of 10-100 Hz were obtained, as shown in Figure S17(b).

Table S7 Natural frequencies of composite laminates with and without metasurface

Order	f_1 (Hz)	f_2 (Hz)	f_3 (Hz)	f_4 (Hz)	f_5 (Hz)
Without MS	25.93	37.53	50.96	66.53	77.67
With MS	23.95	36.62	48.67	62.4	76.45

The corresponding first five natural frequencies are listed in Table S7. The results demonstrate that the integration of the metasurface leads to a certain reduction in the structural natural frequencies; however, the variation of each modal frequency remains below 5 Hz,

indicating that the steel pillar metasurface has a limited influence on the overall dynamic characteristics of the composite structure.

Additionally, the first three mode shapes obtained from the modal tests are presented in Figure S17(c)-(d). Due to the additional mass introduced by the metasurface and slight variations in structural mass distribution and boundary conditions caused by repeated installation processes, certain differences can be observed between the mode shapes of the two configurations. However, the first three modes still correspond to the first-order bending, first-order torsional, and second-order bending modes, respectively, indicating that the overall vibration characteristics remain consistent. The above results demonstrate that the integration of the steel pillar metasurface induces a certain influence on the dynamic characteristics of the composite structure, mainly reflected in limited shifts of the natural frequencies, while the primary vibration modes of the structure remain unchanged.

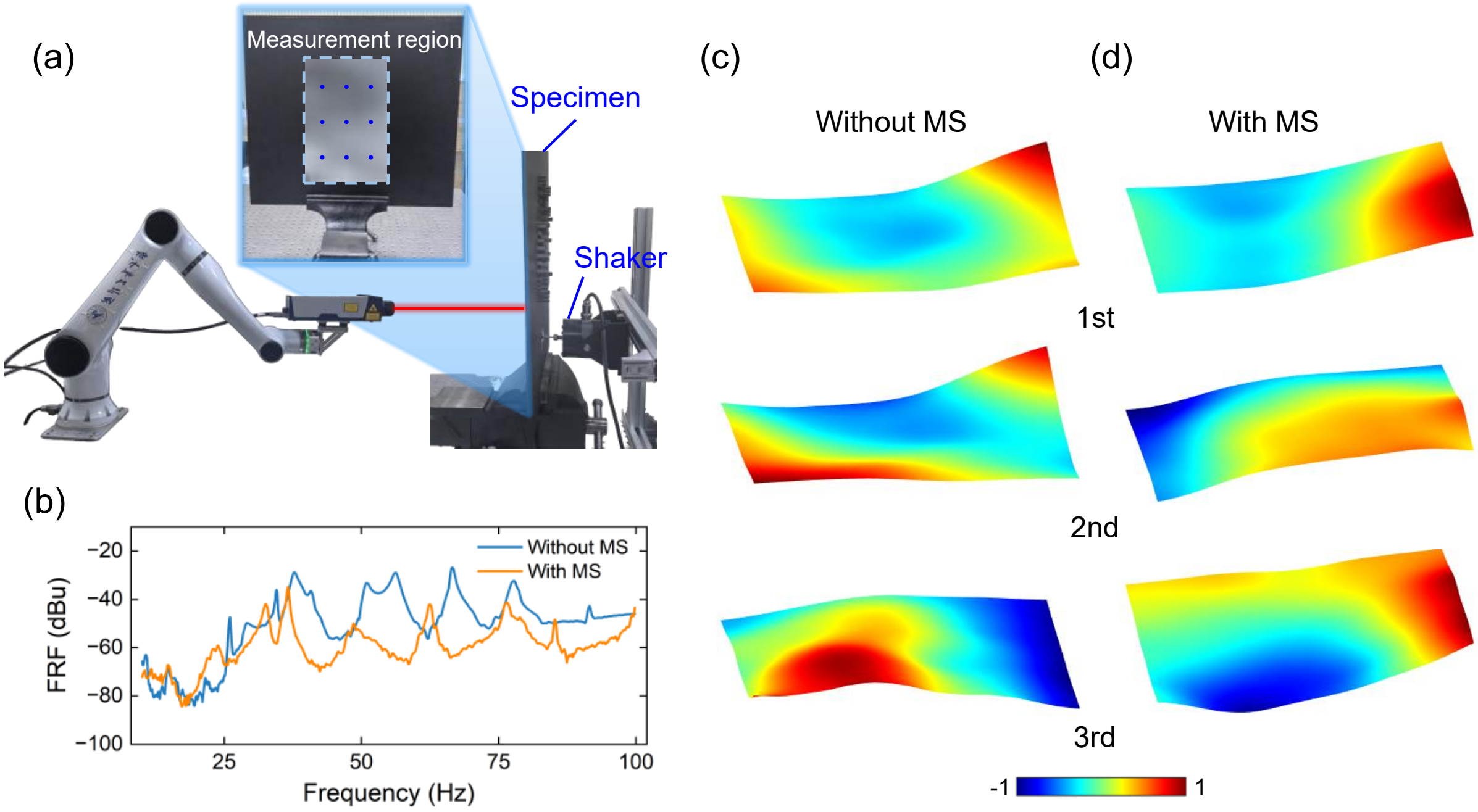

Figure S17. Effect of the metasurface on the dynamic characteristics of the composite laminate structure. (a) Experimental setup for modal testing. (b) Comparison of experimentally measured frequency response curves of the composite laminate with and without the metasurface. (c)-(d) Comparison of the first three experimentally measured mode shapes of the composite laminate with and without the metasurface.
